# Assessing short-term risk of ischemic stroke in relation to all prescribed medications

**DOI:** 10.1038/s41598-021-01115-7

**Published:** 2021-11-04

**Authors:** Imre Janszky, Ioannis Vardaxis, Bo Henry Lindqvist, Jens Wilhelm Horn, Ben Michael Brumpton, Linn Beate Strand, Inger Johanne Bakken, Ingvild Vatten Alsnes, Pål Richard Romundstad, Rickard Ljung, Kenneth Jay Mukamal, Abhijit Sen

**Affiliations:** 1 Department of Public Health and Nursing, Faculty of Medicine and Health Sciences, Norwegian University of Science and Technology (NTNU), Håkon Jarls gate 11 and Mauritz Hanssens gate 2, Trondheim, Norway; 2 Regional Center for Health Care Improvement, St Olav’s Hospital, Trondheim, Norway; 3 Department of Mathematical Sciences, Norwegian University of Science and Technology, Trondheim, Norway; 4 Department of Internal Medicine, Levanger Hospital, Health Trust Nord-Trøndelag, Levanger, Norway; 5 Clinic of Medicine, St. Olav’s Hospital, Trondheim University Hospital, Trondheim, Norway; 6 Department of Public Health and Nursing, K.G. Jebsen Centre for Genetic Epidemiology, Norwegian University of Science and Technology, 7491 Trondheim, Norway; 7 Department of Public Health and Nursing, HUNT Research Centre, Norwegian University of Science and Technology, Levanger, Norway; 8 Department of Health Registries, Norwegian Directorate of Health, Trondheim, Norway; 9 Department of Public Health, University of Stavanger, Stavanger, Norway; 10 Unit of Epidemiology, Institute of Environmental Medicine, Karolinska Institutet, 171 77 Solna, Stockholm Sweden; 11 Department of Medicine, Beth Israel Deaconess Medical Center, Boston, MA USA; 12 Center for Oral Health Services and Research (TkMidt), Trondheim, Norway

**Keywords:** Neurology, Risk factors

## Abstract

We examined the short-term risk of stroke associated with drugs prescribed in Norway or Sweden in a comprehensive, hypothesis-free manner using comprehensive nation-wide data. We identified 27,680 and 92,561 cases with a first ischemic stroke via the patient- and the cause-of-death registers in Norway (2004–2014) and Sweden (2005–2014), respectively, and linked these data to prescription databases. A case-crossover design was used that compares the drugs dispensed within 1 to 14 days before the date of ischemic stroke occurrence with those dispensed 29 to 42 days before the index event. A Bolasso approach, a version of the Lasso regression algorithm, was used to select drugs that acutely either increase or decrease the apparent risk of ischemic stroke. Application of the Bolasso regression algorithm selected 19 drugs which were associated with increased risk for ischemic stroke and 11 drugs with decreased risk in both countries. Morphine in combination with antispasmodics was associated with a particularly high risk of stroke (odds ratio 7.09, 95% confidence intervals 4.81–10.47). Several potentially intriguing associations, both within and across pharmacological classes, merit further investigation in focused, follow-up studies.

## Introduction

Side effects unrecognized at the time of drug approval remain a major concern. As but one example, rofecoxib was approved by the Food and Drug Administration in 1999 and quickly became a best-selling drug worldwide^1^, but subsequent larger trials demonstrated that it increased cardiovascular risk, leading to its withdrawal^2,3^. The example of rofecoxib highlights the limitations of randomized clinical trials used to justify drug approval. Due to their large costs, the trials are usually quite small, often large enough only to be able to detect the expected proximal therapeutic effect. Moreover, these trials are typically short-term and may focus upon direct physiological effects rather than hard clinical outcomes^4^. Thus, these clinical trials may miss rare yet important side effects. Also potentially limiting are the fixed treatment regimens and homogeneous populations that characterize most pre-approval trials, as they disproportionately exclude women, especially in their reproductive age, patients with comorbidities, elderly individuals, and children^5^. The results are often not readily generalizable to the real-life use of medications and to their target patient populations. Finally, poor drug adherence can lead to underestimation of effects of drugs, especially side-effects that may already be uncommon. Thus, a clear need exists for monitoring of pharmaceutical effects of all approved drugs in actual clinical practice.

We have previously conducted a systematic examination of all potential associations between prescribed drugs and short-term risk of acute myocardial infarction^6^ and demonstrated the feasibility of this approach, which we referred to as a “pharmacopeia-wide association study” (PWAS) to emphasize its similarity to genome-wide association studies. In the present study, we extended this approach to examine the short-term risk for ischemic stroke in relation to prescribed medications using comprehensive nation-wide data in two countries.

## Methods

### Study design

We used case-crossover design, a case-only design that compares drug exposure immediately before and more distantly removed from discrete clinical events^7,8^. We specifically included ischemic stroke cases and applied self-matching by comparing drug dispension before the stroke onset with disease-free time in the past as control information. The primary advantage of the case-crossover design is that stable within-person characteristics cannot confound observed associations, enabling the study of acute or triggering effects of transient exposures on outcomes with a sudden onset^9–11^. Because this approach may misestimate the effects of drugs used chronically^12^, it yields estimates that are most reliable for drugs typically taken for short time periods.

### Ascertainment of stroke

We used the Norwegian Patient Registry, the Swedish National Patient Registry, and the cause of death registries in Norway and Sweden to identify cases of ischemic stroke^13,14^. Validation studies show that the quality of information on stroke in these registers, especially in the Norwegian Patient Registry and the Swedish National Patient Register, is very high when the primary diagnosis is used^15,16^. In Norway, all patients with primary ICD-10 hospital discharge diagnoses of I63 from 1 January 2008 to 31 December 2014 were included, as were individuals with the same cause of death from 1 January 2004 to 31 December 2014. In Sweden, the corresponding dates for both the hospital diagnosis and cause of death were between 1 November 2005 and 31 December 2014. For each individual, only the first registered episode of ischemic stroke was included in the analyses.

### Prescribed medications

We assessed the risk of ischemic stroke associated with every drug prescribed to patients that had a first-time stroke within the study period. Data on dispensed medications prior to the event were extracted from the nation-wide registration of dispensed drugs in Norway and Sweden, respectively. The Norwegian Prescription Database was established in 2004^17^. All Norwegian pharmacies are required to supply information on prescriptions including type and dosage of the drug and date of dispensation. Sweden established a similar register, the Swedish Prescribed Drug Register, in 2005^18^. National personal identifiers attached to these data were used to link the information on drug use to other health-related registers existing in these countries. The prescription databases do not include information on drugs purchased over-the-counter or given to institutionalized patients in nursing homes or hospitals. In Norway, it was possible to exclude participants who, at the time of their stroke, were institutionalized and for whom registration of dispensed medications was not available. In Sweden, in the absence of this information, we included only those patients to whom at least one drug was dispensed during the year preceding the occurrence of stroke.

### Statistical analyses

In our primary analysis, for each patient, the occurrence of drug dispensing within 1 to 14 days before the date of ischemic stroke occurrence (case period) was compared to a time window of 29 to 42 days before the ischemic stroke diagnosis (control period) for each drug individually. We included a 14-day wash-out period between the case- and the control-periods to minimize the carryover effects of drugs. These time windows were a priori selected based on the hypothesized hazard periods and the expected induction time for an ischemic stroke^7^. To estimate relative risks, we calculated odds ratios together with 95% confidence intervals, comparing the odds of drug dispensed in the case period to that in the control period using conditional logistic regression.

We assessed all prescribed medications in relation to ischemic stroke risk. Because our aim was to estimate the most likely effect size for drugs with true associations while accounting for simultaneous prescriptions, we opted not to use methods based on simple alpha (i.e., false-positive threshold) penalization to address the problem of multiple comparisons, as it fails to estimate the size of these associations correctly^19^. Instead, we applied a version of the least absolute shrinkage and selection operator (LASSO) regression analysis^19–23^ called BOLASSO (bootstrap-enhanced least absolute shrinkage operator)^24^. With the Bolasso, several bootstrap samples are drawn from the dataset, where each bootstrap sample is generated by sampling N pairs (N is the total number of drugs in the dataset) with replacement. Here, we have drawn 1000 bootstrap samples. Of note, confidence intervals generated via the Bolasso approach are not optimal, because each bootstrap sample is estimated on different penalty parameters, but we include confidence intervals nonetheless for ease of interpretation. However, drugs selected by this approach may include one (i.e., the null) within their confidence intervals. In Bolasso, we obtain multiadjusted estimates as the effect of each selected drug is controlled for the effects of all other selected drugs. In online Supplementary Material, Online Appendix A, we present in detail the background of the method and how we implemented Bolasso in conditional logistic regression models for case-crossover data.

We conducted separate analyses for Norwegian and Swedish data. We present both country-specific and combined estimates for drugs selected by Bolasso from both countries. The combined estimates were calculated using fixed-effect models^25^.

We performed sensitivity analyses to examine the robustness of our results where we extended the case-, control- and wash-out periods from 14 to 30 days (case period = one to 30 days; control period = 61 to 90 days) and repeated all analyses.

All statistical analyses were performed using R (version 3.2.3; R foundation for Statistical Computing, Vienna, Austria) and Stata/IC 16 (Stata Corp, College Station, Texas, USA).

The studies were approved by the Regional Committees for Medical and Health Research Ethics in Central Norway and Regional Ethical Review Board in Sweden. In addition, the use of Norwegian data was also approved by Norwegian Data Protection Authority (Datatilsynet). All data used in the study was anonymised. All methods were performed in accordance with the relevant guidelines and regulations by the respective ethical committees from both Norway and Sweden.

Data used in this research project is available upon request from the respective govermental agencies in Norway and Sweden, respectively.

## Results

Among a total of 120,241 ischemic stroke patients included in the analyses, 92,561 were from Sweden and 27,680 were from Norway. Characteristics of these patients are presented in Table 1.

**Table 1:** Characteristics of the study sample.

	Total N (%)	Sweden N (%)	Norway N (%)
Ischemic stroke patients*	120,241	92,561	27,680
Demise due to ischemic stroke outside hospital	2296 (1.9%)	2201 (2.4%)	95 (0.34%)
Males	55,462 (46.1%)	42,155 (45.5%)	13,307 (48.2%)
**Age (in categories)**			
30–39	204 (0.2%)	1 (0.0%)	203 (0.7%)
40–49	2017 (1.7%)	1383 (1.5%)	634 (2.3%)
50–59	6486 (5.4%)	4613 (5.0%))	1873 (6.8%)
60–69	17,966 (14.9%)	13,361 (14.4%)	4605 (16.6%)
70–79	31,811 (26.5%)	24,541 (26.5%)	7270 (26.3%)
80–89	45,955 (38.2%)	36,044 (38.9%)	9911 (35.8%)
90–99	15,622 (13.0%)	12,478 (13.5%)	3144 (11.4%)
> 100	180 (0.1%)	140 (0.2%)	40 (0.1%)

*The number reflects the patients who were hospitalized or died due to ischemic stroke. In addition, the numbers reflect the patients (N) who dispensed prescribed medicines either in the case-period (1 to 14 days) or control-period (29–42 days) before the date for diagnosis of ischemic stroke (ICD-I63) in Sweden and Norway, respectively.

**Figure 1:**
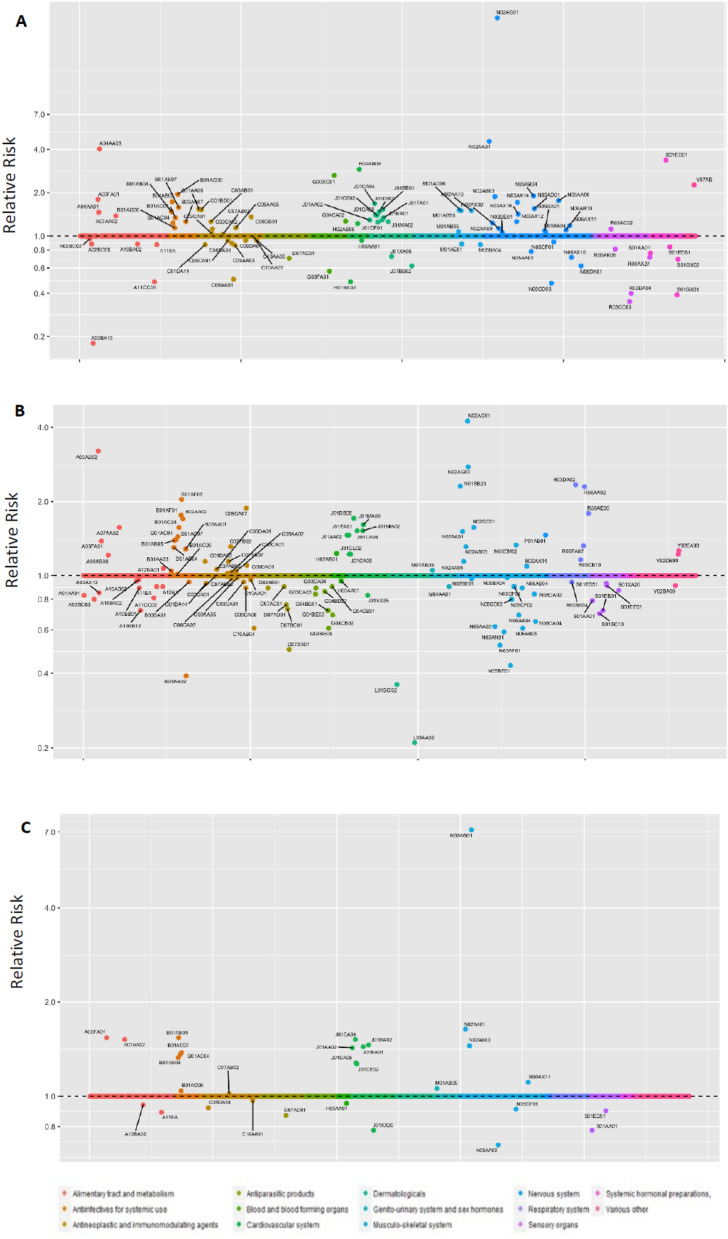
Pharmacopeia-wide association study (PWAS) analyses using pharmaceutical drugs data from Norwegian Prescription Database and Swedish Prescription Drug Register for ischemic stroke. The above plot illustrates (**A**) 102 unique drug types which were selected in Norway, (**B**) 114 unique drug types which were selected in Sweden, and (**C**) 30 drugs which were common hits from both the countries. Y-axis displays relative risk on the log scale. X-axis displays all the drugs studied for a given outcome, grouped by the Anatomical Therapeutic Chemical (ATC) classification.

Out of 1100 prescribed pharmaceutical drugs dispensed for ischemic stroke patients in Norway and 1365 in Sweden, 773 unique drugs were dispensed in either the case- or control- period in Norway and 1141 in Sweden. From these, application of Bolasso selected 102 distinct drugs in Norway and 114 in Sweden. With pooling, a total of 19 drugs were associated with an increased risk for ischemic stroke and 11 drugs with a decreased risk in both countries in these analyses (Fig. 1). Table 2 presents the country-specific and the combined estimates of these mutually-selected drugs.

**Table 2:** Odds ratios for ischemic stroke within 14 days following the drug was dispensed, selected by BOLASSO approach in both countries.

ATC code	Generic names	Sweden			Norway			Total
		Exposed in case period only	Exposed in control period only	OR (95% CI)	Exposed in case period only	Exposed in control period only	OR (95% CI)	Combined estimates OR (95% CI)
**Antibiotics**								
J01AA02	Doxycycline	695	459	1.48 (1.28–1.66)	237	179	1.29 (1.04–1.60)	1.43 (1.28–1.60)
J01CA04	Amoxicillin	475	317	1.46 (1.25–1.71)	268	172	1.66 (1.32–2.09)	1.52 (1.34–1.73)
J01CA08	Pivmecillinam	700	588	1.22 (1.09–1.37)	484	372	1.41 (1.21–1.64)	1.28 (1.17–1.41)
J01CE02	Phenoxymethylpenicillin	744	605	1.22 (1.08–1.37)	380	287	1.40 (1.19–1.65)	1.27 (1.15–1.40)
J01EA01	Trimethoprim	446	309	1.52 (1.29–1.78)	213	162	1.30 (1.04–1.63)	1.44 (1.27–1.64)
J01MA02	Ciprofloxacin	826	547	1.52 (1.34–1.72)	216	154	1.26 (0.99–1.60)	1.46 (1.31–1.63)
J01XX05	Methenamine	578	627	0.83 (0.67–1.04)	320	383	0.72 (0.56–0.93)	0.78 (0.66–0.92)
S01AA01	Choramphenicol	194	242	0.79 (0.63–0.99)	166	222	0.78 (0.63–0.96)	0.78 (0.66–0.91)
**Antifungal agent**								
A07AA02	Oral nystatin	270	170	1.57 (1.28–1.94)	104	64	1.38 (0.95–2.01)	1.52 (1.27–1.83)
**Antithrombotic agents**								
B01AB04	Dalteparin	597	459	1.30 (1.10–1.53)	147	111	1.49 (1.05–2.10)	1.33 (1.15–1.55)
B01AB05	Enoxaparin	126	101	1.39 (0.97–1.98)	124	73	1.73 (1.17–2.56)	1.54 (1.18–1.99)
B01AC04	Clopidogrel	1265	1018	1.40 (1.25–1.57)	328	292	1.24 (1.01–1.52)	1.36 (1.23–1.50)
B01AC06	Acetylsalicyclic acid	18,467	18,667	1.01 (0.98–1.05)	4461	4411	1.14 (1.07–1.21)	1.04 (1.01–1.07)
B01AC07	Dipyridamole	410	298	1.44 (1.22–1.70)	500	446	1.33 (1.13–1.57)	1.38 (1.23–1.56)
**Anti-inflammatory drug**								
M01AB05	Diclofenac	1191	1141	1.05 (0.96–1.15)	454	443	1.06 (0.91–1.23)	1.06 (0.98–1.14)
**Propulsive and anti-emetic drug**								
A03FA01	Metoclopramide	549	397	1.38 (1.17–1.64)	417	243	1.81 (1.48–2.22)	1.54 (1.35–1.76)
**Anti-diabetic drug**								
A10BA02	Metformin	3062	3247	0.96 (0.89–1.04)	887	985	0.88 (0.77–1.01)	0.94 (0.88–1.01)
**Anti-thyroid agent**								
H03AA01	Levothyroxine sodium	5675	5831	0.95 (0.89–1.02)	1058	1111	0.94 (0.83–1.05)	0.95 (0.89–1.01)
**Antipsychotics**								
N05AA02	Levomepromazine	269	307	0.62 (0.42–0.92)	114	127	0.78 (0.55–1.12)	0.70 (0.54–0.92)
**Antidepressant**								
N06AX11	Mirtazapine	3018	2998	1.09 (0.97–1.23)	294	284	1.17 (0.93–1.48)	1.11 (1.00–1.23)
**Antiglaucoma in combination with beta-blocker**								
S01ED51	Timolol, combinations	751	833	0.93 (0.83–1.04)	253	300	0.83 (0.69–1.01)	0.90 (0.82–1.00)
**Beta blocking agent**								
C07AB02	Metoprolol	11,540	11,596	1.02 (0.97–1.06)	3584	3595	1.02 (0.95–1.10)	1.02 (0.98–1.06)
**Corticosteriods, dermatologicals**								
D07AC01	Betametason	394	454	0.90 (0.78–1.05)	70	101	0.71 (0.50–1.01)	0.87 (0.76–1.00)
**Hypnotics and sedatives**								
N05CF01	Zopiclone	6046	6312	0,90 (0,85–0,96)	2266	2471	0,92 (0,84–0,99)	0,91 (0,86–0,95)
**Opioids**								
N02AA01	Morphine	1316	1048	1.51 (1.30–1,76)	165	77	4.53 (2.67–7.68)	1.64 (1.42–1.90)
N02AB03	Fentanyl	392	318	1.31 (1.07–1.61)	189	96	1.87 (1.35–2.60)	1.45 (1.22–1.72)
N02AG01	Morphine and antispasmodics	121	35	4.24 (2.71–6.64)	46	0	33.99 (15.56–74.26)	7.09 (4.81–10.47)
**Lipid modifying agent**								
C10AA01	Simvastatin	6672	6871	0.96 (0.91–1.01)	2364	2401	0.99 (0.92–1.07)	0.97 (0.93–1.01)
**Vasodilators**								
C01DA14	Isosorbide mononitrate	4469	4632	0.93 (0.86–1.01)	801	875	0.87 (0.74–1.02)	0.92 (0.85–0.99)
**Vitamins**								
A11EA	Vitamin B-complex, plain	1941	2052	0.90 (0.80–1.02)	845	947	0.88 (0.76–1.02)	0.89 (0.81–0.98)

All generic names listed according to the Anatomical Therapeutic Chemical (ATC), 5th level.

Case crossover analysis, case period (1–14 days) and control period (29–42 days) before the index-date for the diagnosis of ischemic stroke.

### Cardiovascular drugs

Several antithrombotic agents and metoprolol were associated with elevated stroke risk. Other cardiovascular drugs, like simvastatin and isosorbide mononitrate, were associated with lower risk for ischemic stroke.

### Antibiotics/antifungal agents

We observed an increased risk for ischemic stroke in association with the use of doxycycline, amoxicillin, pivmecillinam, phenoxymethylpenicillin, trimethoprim, ciprofloxacin, and oral nystatin. On the other hand, methenamine and chloramphenicol were associated with a lower risk.

### Analgesics

Several opioid analgesics, especially morphine in combination with antispasmodics were associated with higher risk of stroke. Among non-opioid agents, diclofenac was also associated with a slightly increased risk for stroke.

### Psychoactive medications

Mirtazapine was associated with an increased while levomepromazine and zopiclone with a decreased risk for ischemic stroke.

### Other medications

Metoclopramide was associated with an elevated risk for ischemic stroke. In contrast, metformin, levothyroxine, vitamin B-complex, betamethasone and timolol used against glaucoma were associated with a lower risk for ischemic stroke.

In online supplementary material, in Tables S1 and S2, we present estimates for all drugs selected by Bolasso in either Norway or Sweden, respectively.

### Sensitivity analyses

In Table 3, we present the results of our analyses where we extended the case-, control- and wash-out periods from 14 to 30 days. The point estimates were generally comparable to those in our main analyses. These analyses selected slightly more drugs, and we observed an increased risk for ischemic stroke for nitrofurantoin, ticagrelor, apixaban, sumatriptan, ferrous sulfate, macrogol, diazepam, oxazepam, escitalopram, prednisolone, furosemide, spironolactone, clomethiazole, buprenorphine, tramadol, and for oxycodone alone and when used in combination with naloxone. Some additional drugs, such as warfarin, cromoglicic acid, and latanoprost, were associated with lower stroke risk. In Supplementary Tables S3 and S4, we present all drugs selected by Bolasso in these sensitivity analyses in either Norway or Sweden, respectively.

**Table 3:** Odds ratio for ischemic stroke within 30 days following the drug was dispensed, selected by BOLASSO approach in both countries.

ATC Code	Generic names	Sweden			Norway			Total
		Exposed in case period only	Exposed in control period only	OR (95% CI)	Exposed in case period only	Exposed in Control period only	OR (95% CI)	Combined estimates OR (95% CI)
**Antibiotics**								
J01AA02	Doxycycline	1224	951	1.26 (1.15–1.38)	472	341	1.36 (1.16–1.59)	1.28 (1.19–1.39)
J01CA04	Amoxicillin	848	682	1.17 (1.04–1.31)	472	330	1.37 (1.17–1.62)	1.23 (1.12–1.36)
J01CA08	Pivmecillinam	1380	1124	1.21 (1.11–1.32)	885	739	1.21 (1.09–1.35)	1.21 (1.13–1.29)
J01CE02	Phenoxymethylpenicillin	1445	1231	1.13 (1.04–1.31)	688	548	1.25 (1.10–1.41)	1.17 (1.09–1.25)
J01MA02	Ciprofloxacin	1514	1153	1.23 (1.12–1.35)	386	278	1.31 (1.09–1.58)	1.25 (1.15–1.35)
J01XE01	Nitrofurantoin	776	631	1.28 (1.13–1.45)	262	201	1.25 (0.99–1.57)	1.27 (1.14–1.42)
**Antifungal agent**								
A07AA02	Oral nystatin	452	331	1.28 (1.08–1.51)	177	118	1.37 (1.04–1.82)	1.30 (1.13–1.51)
**Antithrombotic agents**								
B01AA03	Warfarin	3323	3406	0.92 (0.86–0.97)	1546	1606	0.99 (0.91–1.08)	0.94 (0.90–0.99)
B01AB04	Dalteparin	1155	807	1.45 (1.27–1.67)	261	173	1.70 (1.25–2.30)	1.49 (1.31–1.69)
B01AB05	Enoxaparin	251	196	1.27 (0.98–1.66)	224	141	1.46 (1.07–2.00)	1.35 (1.10–1.65)
B01AC04	Clopidogrel	2433	2067	1.27 (1.16–1.39)	660	535	1.43 (1.21–1.68)	1.31 (1.21–1.41)
B01AC06	Acetylsalicyclic acid	38,514	37,348	1.03 (1.00–1.06)	9116	8712	1.11 (1.06–1.16)	1.05 (1.03–1.08)
B01AC07	Dipyridamole	731	574	1.33 (1.17–1.51)	994	916	1.22 (1.07–1.40)	1.28 (1.16–1.40)
B01AC24	Ticagrelor	122	50	2.96 (1.87–4.66)	39	19	2.49 (1.02–6.06)	2.86 (1.90–4.29)
B01AF02	Apixaban	51	25	2.41 (1.25–4.65)	26	13	3.15 (0.93–10.6)	2.56 (1.44–4.56)
**Analgesics**								
N02BE01	Paracetamol	28,071	26,895	1.04 (1.01–1.08)	4112	3681	1.09 (1.01–1.17)	1.05 (1.02–1.08)
N02CC01	Sumatriptan	199	151	1.45 (1.05–2.00)	91	70	1.52 (0.94–2.46)	1.47 (1.13–1.92)
**Anti-inflammatory drugs**								
M01AB05	Diclofenac	2389	2214	1.10 (1.02–1.18)	876	761	1.15 (1.02–1.30)	1.11 (1.05–1.35)
**Anti-allergics**								
S01GX01	Cromoglicic acid	143	192	0.73 (0.56–0.95)	30	44	0.62 (0.35–1.12)	0.71 (0.56–0.90)
**Anti-anemic agent**								
B03AA07	Ferrous sulfate	4614	4235	1.23 (1.12–1.35)	732	664	1.43 (1.12–1.83)	1.25 (1.15–1.37)
**Anti-emetic and propulsives**								
A03FA01	Metoclopramide	1009	669	1.46 (1.27–1.67)	707	481	1.50 (1.27–1.77)	1.48 (1.33–1.64)
**Antidiabetic drug**								
A10BA02	Metformin	6451	6635	0.95 (0.90–1.01)	1896	2031	0.91 (0.83–1.01)	0.94 (0.89–0.99)
**Anti-constipation drug**								
A06AD65	Macrogol, combinations	1879	1616	1.06 (0.98–1.14)	62	34	1.58 (0.98–2.55)	1.07 (0.99–1.15)
**Antipsychotics**								
N05AA02	Levomepromazine	607	642	0.67 (0.48–0.93)	248	266	0.74 (0.54–1.00)	0.71 (0.56–0.89)
**Anxiolytics**								
N05BA01	Diazepam	2021	1931	1.14 (1.01–1.27)	1552	1386	1.15 (1.03–1.29)	1.15 (1.06–1.24)
N05BA04	Oxazepam	9180	8827	1.15 (1.08–1.23)	2075	1933	1.16 (1.04–1.29)	1.15 (1.09–1.22)
**Antidepressants**								
N06AB10	Escitalopram	1179	1112	1.21 (1.00–1.47)	1259	1165	1.13 (0.98–1.29)	1.16 (1.03–1.29)
N06AX11	Mirtazapine	6333	5897	1.36 (1.23–1.49)	629	566	1.26 (1.02–1.55)	1.34 (1.23–1.46)
**Antiglaucoma medications**								
S01EE01	Latanoprost	1753	1847	0.93 (0.86–1.01)	531	566	0.89 (0.76–1.04)	0.92 (0.86–0.99)
**Beta blocking agent**								
C07AB02	Metoprolol	24,140	22,771	1.07 (1.04–1.11)	7389	7048	1.08 (1.02–1.14)	1.07 (1.04–1.10)
**Corticosteroids for systemic use**								
H02AB06	Prednisolone	6122	5848	1.09 (1.02–1.17)	1811	1548	1.22 (1.10–1.35)	1.13 (1.07–1.20)
**Diuretics**								
C03CA01	Furosemide	29,041	27,956	1.08 (1.04–1.12)	3742	3638	1.10 (1.02–1.20)	1.08 (1.05–1.12)
C03DA01	Spironolactone	5781	5407	1.13 (1.05–1.22)	657	575	1.23 (1.02–1.48)	1.14 (1.07–1.23)
**Hypnotics and sedatives**								
N05CM02	Clomethiazole	1076	923	1.64 (1.33–2.03)	88	46	4.13 (1.87–9.15)	1.74 (1.42–2.14)
**Lipid modifying agent**								
C10AA01	Simvastatin	13,949	13,753	0.97 (0.94–1.01)	4911	4882	0.96 (0.91–1.02)	0.97 (0.94–1.00)
**Opioids**								
N02AA01	Morphine	2533	1954	1.66 (1.46–1.88)	252	117	3.03 (2.04–4.52)	1.75 (1.56–1.98)
N02AA05	Oxycodone	4285	3571	1.34 (1.21–1.48)	604	474	1.35 (1.06–1.72)	1.34 (1.22–1.47)
N02AA55	Oxycodone and naloxone	208	62	2.05 (1.17–3.60)	53	31	2.18 (0.91–5.22)	2.09 (1.30–3.35)
N02AB03	Fentanyl	809	696	1.45 (1.21–1.75)	319	162	2.62 (1.77–3.89)	1.61 (1.37–1.91)
N02AE01	Buprenorphine	949	843	1.22 (1.06–1.40)	406	358	1.17 (0.93–1.47)	1.21 (1.07–1.36)
N02AG01	Morphine and antispasmodics	167	62	3.65 (2.45–5.44)	49	1	15.70 (6.01–41.02)	4.52 (3.13–6.54)
N02AX02	Tramadol	4062	3993	1.04 (0.97–1.11)	1286	1026	1.35 (1.18–1.54)	1.10 (1.03–1.17)
**Vasodilators**								
C01DA14	Isosorbide mononitrate	9485	9368	0.97 (0.91–1.03)	1725	1708	0.95 (0.84–1.06)	0.97 (0.91–1.02)

All generic names listed according to the Anatomical Therapeutic Chemical (ATC), 5th level.

Case crossover analysis, case-period (1–30 days) and control period (61–90 days) before the index-date for the diagnosis of first ischemic stroke.

## Discussion

It has been difficult to identify drugs that may influence ischemic stroke risk because of its relative rarity and complex physiology. To the best of our knowledge, this is the first study to systematically examine all possible associations between pharmaceutical drugs requiring a prescription and short-term risk for ischemic stroke. Ultimately, we identified 19 drugs that were consistently associated with increased stroke risk in both Norway and Sweden in our main analyses. Similarly, eleven drugs were consistently associated with a lower risk in both countries.

Several cardiovascular drugs, especially antithrombotics, were associated with an elevated short-term risk for stroke. These associations are most likely explained by the indications for these drugs, although differences within the same group of medications might indicate differential effects. The vasodilator isosorbide mononitrate had an inverse association, which is intriguing since it is given as a symptomatic treatment for acute coronary heart disease, i.e., to patients who are clearly at increased risk for ischemic stroke. Many of the other medications provided to these patients were either not selected or were associated with an increased risk. The inverse association observed for a chronically used drug like simvastatin may reflect the adverse effect of the discontinuation of the drug use^26,27^.

Opioids were found to be associated with an increased short-term risk for ischemic stroke. This increase was particularly strong for morphine in combination with antispasmodics, which had the strongest association with ischemic stroke in both our main and secondary analyses. Interestingly, we found the same in our previous PWAS of myocardial infarction, with a relative risk of six^6^. Although we cannot establish causality, opioids might affect stroke risk directly for example via decreased oxygenation^28^. It is not clear why there was a markedly increased risk for the combination of morphine with antispasmodics, but these combined analyses raise important questions about the continued availability of this combination.

Several antibiotics and the antifungal nystatin were associated with an increased risk for ischemic stroke. This might reflect the indication of these medications, i.e., infectious diseases may trigger cardiovascular events, including stroke^29,30^. However, as far from all antibiotics were associated with increased stroke risk, it is not clear whether the selected drugs have indications that are particularly strong triggers or these drugs have physiological effects increasing the probability of a stroke. Ampicillin which had the strongest association with stroke risk among the antibiotics might increase the risk of a thrombus formation by interacting with warfarin^31^. Chloramphenicol and methenamine were associated with a decreased risk both in Sweden and Norway. Their main indications, i.e., eye and urinary infections, respectively, are unlikely to be protective against a stroke. We found no previous studies assessing the association of chloramphenicol and methenamine with stroke risk nor can we explain our findings based on the known physiological effects of these drugs. Thus, the inverse association seen in the case of these two drugs needs evaluation in subsequent studies. If there is any true protection from these drugs, it may last only for a very short time as these drugs were not selected in our sensitivity analyses when we extended the exposure windows.

Among psychoactive drugs, atypical antidepressant mirtazapine was associated with an increased risk for ischemic stroke. In contrast, neuroleptic levomepromazine and hypnotic zopiclone were associated with a decreased risk. In secondary analyses, where we extended the exposure windows, some benzodiazepines and the selective serotonin uptake inhibitor escitalopram were also associated with an increased risk, but zopiclone was not selected. Antidepressant use has been associated with an increased risk of stroke in previous studies^32^, as have the use of antipsychotics^33,34^. Thus, it is intriguing that we observed an inverse association for levomepromazine. This drug has a complex biological activity and it has effects on a wide range of different receptors^35^.

We found no previous studies examining the association of metoclopramide with stroke risk and we cannot readily explain the consistently elevated risk observed both in the main and the sensitivity analyses by the known physiological effects or the indications of the drug. However, we hypothesize that insufficient blood flow in the area of the arteria cerebri posterior and the resulting diplopia, reduced vision, and dizziness with nausea may be the indication for the use of this drug. The inverse association for metformin and vitamin B complex was expected based on prior studies^36,37^. In contrast, we found no previous studies on stroke risk and levothyroxine, timolol, and betamethasone, which all demonstrated inverse associations in our main analyses.

### Strengths and limitations

We performed nation-wide studies in Sweden and Norway examining all prescribed medications in relation to short-term risk for stroke. Given the size of these countries and the length of the follow-up time, we had considerable statistical power and generally estimated relative risks with high precision. The health care systems in these countries are universal and equally accessible to virtually all the residents. Participation in the fully digitalized health registers used in this study was mandatory. Thus, biased recall or self-selection is avoided in our study. Also, the quality of the information in these registers is generally high^15,16^. Furthermore, our results are unlikely to be confounded by stable patient characteristics, chronic conditions, or lifestyle-related factors associated with medication use and influencing stroke risk as we applied self-matching^7^.

Besides its strengths, our study also had limitations. We conducted a large screening of possible hypotheses and as in any similar hypothesis-free settings, like in GWA studies, the results should be interpreted with caution. We took into account the problem of multiple comparisons by the robust Bolasso method, but our results should generally be confirmed in focused studies before any specific drug is recommended or discouraged.

Given the explorative nature of our work, we uniformly analyzed all drugs and consequently, the hypothesized case-, control- and wash-out periods might not be optimal for some drugs. However, it is important to recognize that such uncertainties do not lead to overestimation of effects in a case-crossover study^7,8^ and when we extended these periods, in our sensitivity analyses, we generally got similar results. Also, case-crossover studies are prone to the so-called ‘persistent user bias’^12^, which might lead to an upward bias of the estimates in case of chronically-used drugs. In this study, we could not differentiate between acute and chronic use and therefore caution is needed when interpreting findings for drugs used chronically. However, persistent user bias is not likely to explain the observed protective effects, nor the differences observed within classes of drugs.

Case-crossover studies are not immune to confounding by time-varying characteristics. Most relevantly, as we emphasized above, the effect of a drug and its indication were not directly separable in our study. Consequently, it was often not clear whether the observed effects were due to the drugs or due to the conditions the drugs were prescribed for. However, markedly different associations with ischemic stroke within the same drug class might have indicated a direct effect for certain drugs. Finally, the prescription databases do not contain information on the actual date of self-administration of drugs, only on date of dispension which would be expected to produce non-differential misclassification and a bias toward the null.

In conclusion, this pharmacopeia-wide association study demonstrates the feasibility of a national, universal approach to identifying drugs that may trigger, or protect against ischemic stroke. Several potentially intriguing associations, both within and across pharmacological classes, merit further investigation in focused, follow-up studies.

## Supplementary Information

Supplementary Information.

Supplementary Tables.
