# Supplementary material for: Assessing short-term risk of ischemic stroke in relation to all prescribed medications
Source: Sci Rep. 2021 Nov 4;11:21673. doi: 10.1038/s41598-021-01115-7 (PMC8568938; doi:10.1038/s41598-021-01115-7)
Supplement: Supplementary file 1 — Supplementary Information. [file 41598_2021_1115_MOESM1_ESM.pdf]

Appendix A

The case-crossover design

A.1 The likelihood function under the case-crossover design

In our analysis, the subjects are the patients who experienced the event (case), and each patient is both the case and the control of itself, in different time periods. For the whole analysis, we used one case and one control window for each patient. According to [6], our design is called a non-localizable design and, more specifically, an unidirectional design, because the windows are placed after observing the event for each patient and not a priori.

Assume that we have a total of N individuals who experienced the event at some point in the time set $\{1, ..., T\}$. Here, T is the length of the observation period, and the $t \in \{1, ..., T\}$ are the discrete time points where each case was checked (followed up). Let Y_{it} be the risk occurrence indicator for subject i at time t, that is $Y_{it} = 1$ if the event took place at time t and $Y_{it} = 0$ otherwise. For $i = 1, \ldots, N$ and $t = 1, \ldots, T$, let $X_{it} = (X_{it1}, ..., X_{itp})$ be the p-dimensional vector of exposures at time t for the ith subject. Let further $X_i = (X_{it}; t = 1, ..., T)$ be the corresponding p-dimensional exposure time series vector.

Then, if it is assumed that events follow a proportional hazards model, and if it is assumed that events are rare, we will assume that

$$P\left(Y_{it} = 1 | X_i\right) = \lambda_{0it} e^{X_{it}\beta}, \tag{1}$$

where $\beta = (\beta_1, ...\beta_p)'$ is a vector of coefficients. The baseline function λ_{0it} is assumed to vary among subjects, carrying characteristics of the ith subject like, for example, age, or other characteristics which may change with time t. In other words, Eq. (1) is the probability that the ith subject will experience the event exactly at time t, given its exposure vector and characteristics.

Assume first that the exposure of each subject is observed throughout the time set $\{1, 2, ..., T\}$. Then by conditioning on the occurrence of exactly one event in $\{1, 2, ..., T\}$, and as above assuming that events are rare, the contribution to the likelihood from the ith subject would be ([8, 6]):

$$\begin{aligned}
& P\left(Y_{i,t_i} = 1 \middle| \sum_{s=1}^{T} Y_{is} = 1, X_i\right) \\
&= \frac{P\left(Y_{i,t_i} = 1 | X_i\right)}{P\left(\sum_{s=1}^{T} Y_{is} = 1 | X_i\right)} \\
&= \frac{P\left(Y_{i,t_i} = 1 | X_i\right)}{\sum_{s=1}^{T} P\left(Y_{is} = 1 | X_i\right)}
\end{aligned}$$

$$= \frac{\lambda_{0it_i} e^{X_{it_i}\beta}}{\sum_{s=1}^{T} \lambda_{0is} e^{X_{is}\beta}} \tag{2}$$

The complete likelihood is then the product of these contributions, namely

$$L(\beta) = \prod_{i=1}^{N} \frac{\lambda_{0it_i} e^{X_{it_i}\beta}}{\sum_{s=1}^{T} \lambda_{0is} e^{X_{is}\beta}}. \tag{3}$$

Since we do not know the exposure X_{it} for all $t \in 1, 2, ..., T$, but only in a referent window W_i, following Lumley and Levy [8], we replace the denominators in Eq. (3) by the simplification where s runs only in the referent window. Thus, we replace Eq. (3) by

$$L(\beta) = \prod_{i=1}^{N} \frac{\lambda_{0it_i} e^{X_{it_i}\beta}}{\sum_{t \in W_i} \lambda_{0it} e^{X_{it}\beta}} = \prod_{i=1}^{N} \frac{e^{X_{it_i}\beta}}{\sum_{t \in W_i} e^{X_{it}\beta}} = \prod_{i=1}^{N} \frac{e^{X_{it_i}\beta}}{e^{X_{it_i}\beta} + e^{X_{is_i}\beta}}. \tag{4}$$

In our application, $W_i = \{t_i, s_i\}$, where t_i and s_i are the case and the control windows of the ith subject, respectively. Furthermore, the λ_{0it_i} and λ_{0it} are cancelled because they are assumed to be (approximately) constant in the referent windows. Now, X_{it_i} is a vector of length p corresponding to the exposure of each drug in the case window, while X_{is_i} is a vector of the same length corresponding to the exposures in the control window. Both vectors contain the values 0 and 1 for non-exposure and exposure, respectively.

It should be noted that Eq. (4) is not an actual likelihood because of the reduction of the sum in the denominator of (3). Thus, as noted by Lumley and Levy [8], the resulting estimates may be biased. This bias, called overlap bias, will however usually be small (see [8]).

On the other hand, Eq. (4) has the form of the likelihood of a conditional logistic regression (CLR) and can hence be analyzed using standard statistical packages allowing CLR, Note that the window W_i in Eq. (4) consists of two time points, t_i (case) and s_i (control), while more controls can be included by adding points in the window.

A.2 Generalized linear model

Our model can be represented differently in order to show that it is a generalized linear model of logistic type. Consider the matrices $X_t = (X_{1t_1}, ..., X_{Nt_N})^T$, corresponding to the case windows, and $X_s = (X_{1s_1}, ..., X_{Ns_N})^T$, corresponding to the control windows, both of dimension $N \times p$, where X_{it_i} and X_{is_i} are defined above. Consider also the corresponding response vectors $Y_t = (Y_{1t_1}, ..., Y_{Nt_N})^T$ and $Y_s = (Y_{1s_1}, ..., Y_{Ns_N})^T$, for the cases and controls, respectively. These are both N-dimensional, where the response vector for the cases contains only the element 1 and the response vector for the controls contains only the element 0. This is because we restrict attention to patients with exactly one event in the

given time set. The likelihood $L(\beta)$ can hence be written in the form

$$L(\beta) = \prod_{i=1}^{N} \frac{e^{X_{it_i}\beta}}{e^{X_{it_i}\beta} + e^{X_{is_i}\beta}} = \prod_{i=1}^{N} \frac{1}{1 + e^{-X_i\beta}}, \tag{5}$$

where $X_i = X_{it_i} - X_{is_i}$, that is, we subtract the control vector from the case vector for each patient. As will be seen below, for a case-crossover design with one case and one control window, the likelihood can be written in the form of the unconditional likelihood for a binary logistic regression with no intercept and constant response equal to one (see Avalos et al.[3]). The fact that the response is constant and equal to one, becomes more clear when we subtract the response vector Y_s from Y_t. Note that now the data matrix X consists of values $-1, 0, 1$. Furthermore, for a specific component β_j of the vector β, e^{β_j} is the relative risk for the event, if the jth component of X_i is increased by 1, which means that the corresponding medicine is taken in the case window and not in the control window.

By letting p_i be the contribution of the ith individual to the likelihood in Eq. (5), that is $p_i = \frac{1}{1+e^{-X_i\beta}} = \frac{e^{X_i\beta}}{1+e^{X_i\beta}}$, then the likelihood can be written in the following form:

$$L(\beta) = \prod_{i=1}^{N} \frac{1}{1 + e^{-X_i\beta}} = \prod_{i=1}^{N} p_i = \prod_{i=1}^{N} p_i^{y_i} (1 - p_i)^{1-y_i}, \tag{6}$$

since $y_i = 1$ for all i. The log-likelihood is of the form

$$\begin{aligned}
\ell(\beta) &= \log(L(\beta)) = \sum_{i=1}^{N} \log\left[p_i^{y_i}(1-p_i)^{1-y_i}\right] \\
&= \sum_{i=1}^{N} \left[y_i \log(p_i) + (1 - y_i)\log(1 - p_i)\right] \\
&= \sum_{i=1}^{N} \left[y_i \text{logit}(p_i) + \log(1 - p_i)\right],
\end{aligned} \tag{7}$$

which corresponds to the likelihood of logistic regression ([9, 10])

Finally, the log-likelihood function given in Eq. (7) is a concave function with respect to the β coefficients. This can easily be seen by rewriting the function in the following form,

$$\begin{aligned}
\ell(\beta) &= \sum_{i=1}^{N} \left[y_i \text{logit}(p_i) + \log(1 - p_i)\right] \\
&= \sum_{i=1}^{N} \left(y_i X_i\beta + \log\left(\frac{1}{1 + e^{X_i\beta}}\right)\right) \\
&= \sum_{i=1}^{N} \left(y_i X_i\beta - \log\left(1 + e^{X_i\beta}\right)\right)
\end{aligned}$$

and noting that $-\log(1 + e^x)$ is a concave function.

A.3 The lasso method and glmnet

In this section we describe how we estimated the coefficients in β using the lasso (the least absolute shrinkage and selection operator) method ([11]). More specifically, we estimated the coefficients under the lasso penalty by minimizing the negative penalized log-likelihood in Eq. (7), that is,

$$\hat{\beta}(\lambda) = \text{argmin}_{\beta} \left(-\ell(\beta) + \lambda \sum_{j} |\beta_j| \right).$$

The estimation was done via a coordinate descent algorithm using the glmnet package ([5]) in the statistical package R. We chose the binomial family with logit link function in the settings of the glmnet function. Furthermore, the choice of λ was done by generating a sequence of 100 λ values and then choosing the best, using 10-fold cross-validation. All of those functions are provided by the package.

The glmnet function for the binomial model requires a two-level response. In our case we have, however, a constant response equal to one. To overcome this problem and allow the use the glmnet function, we added N rows of zeros to the data matrix X, and N new observations to the response vector Y, all with the value zero. We demonstrate below that this does not change the likelihood function, and since the response vector now contains both zeros and ones, the estimation of β can be done using the glmnet function,

To see why the above claim is true, let j correspond to lines of the extended data matrix X which were added, while i corresponds to lines of the original data matrix. Then $p_j = 1/(1+e^0) = 1/2$, while $p_i = \frac{1}{1+e^{-X_i\beta}}$, as before. Hence the likelihood in Eq. (6), using the extended data matrix, becomes

$$L(\beta) = \prod_{j=1}^{N} p_j^{y_j}(1-p_j)^{1-y_j} \prod_{i=1}^{N} p_i^{y_i}(1-p_i)^{1-y_i} = \prod_{j=1}^{N}(1-p_j)\prod_{i=1}^{N} p_i = (1/2)^N \prod_{i=1}^{N} p_i, \tag{8}$$

which equals the original likelihood in Eq. (6) multiplied by a constant independent of the parameters. Hence, the estimated coefficients are not affected by the modification. We chose N additional observations with zero response because, as we will discuss in Appendix A.4, we use bootstrapping to sample from the data matrix X. We found that N extra observations are enough to ensure both 0 and 1 responses in each bootstrap sample. If we alternatively added, say, only one extra observation, we would most probably get some bootstrap samples with only 1 as a response, and thus the glmnet function would not work. However, as already noted, the actual number of extra observations with zero response does generally not affect the estimated coefficients.

A.4 Bolasso

Lasso sometimes allows a few irrelevant variables to enter the model ([3]). Further, according to Tibshirani [11], the standard errors for the lasso estimates

are difficult to acquire. Those problems can be reduced by bootstrapping. Tibshirani [11], Avalos et al. [3] and Avalos et al. [2] suggested a bootstrap version of lasso which is called bolasso, which reduces the uncertainties of the lasso estimates. Bolasso runs a bootstrap on many samples, and estimates the parameters for each sample, using the lasso approach.

In our analysis we ran a bootstrap on the lasso model described in Section A.3, using 1000 bootstrap samples. For each bootstrap sample, a new λ sequence was generated by the glmnet package and the optimal λ was chosen using 10-fold cross validation. Then the model was fitted by glmnet using the current bootstrap sample.

The β-coefficients frequently selected by the lasso from the bootstrap samples are taken into account, while the others are set to zero [3]. Avalos et al. [3] suggested using Akaike's information criterion ([1]), $AIC = -2\ell(\hat{\beta}) + 2k$, for estimating the frequency threshold of bolasso. Here $\ell(\hat{\beta})$ is the log-likelihood function applied on the estimated coefficients from each model under selection, and k is the total number of non-zero elements in the β vector. AIC corresponds to an estimation of the allowed number of times that a coefficient could have been set to zero among the bootstraps. We can compute the AIC for different models and then choose the model which gives the lowest AIC-value.

The output from the bolasso in our analysis is a matrix of dimension $p \times B$. Each column of the matrix corresponds to one bootstrap replicate, that is, the estimated vector of coefficients from that bootstrap sample. Each row of the matrix corresponds to the values that the specific coefficient took among the bootstrap samples. For each row in the matrix, we computed the total number of zeros and we created a frequency vector of length p. For each value in the frequency vector we created a model by checking which of the coefficients had been set to zero less times than the chosen threshold. For those, their mean among the bootstraps was taken as an estimate, that is

$$\hat{\beta}_j = \sum_{b=1}^{B} \beta_j^{*b} / B,$$

where β_j^{*b} is the b-th bootstrap estimate of coefficient β_j and B is the total number of bootstrap replicates ([4]). The other coefficients are simply set to zero. Next, the AIC was computed for the corresponding model, where k is the number of non-zero estimates in the newly computed β vector. Finally, this is done for all thresholds in the sequence and the one which gives the lowest AIC is chosen as optimal. For that optimal threshold we again check which of the coefficients have been set to zero less times than the optimal threshold and we compute $\hat{\beta}_j$ as before, while the others are set to zero. This vector of coefficients is the bolasso estimate of the coefficients for the objective function ([3]).

Finally, we can compute the standard error of each coefficient as

$$\hat{SD}_j = \left(\sum_{b=1}^{B} \left(\beta_j^{*b} - \hat{\beta}_j \right)^2 / (B-1) \right)^{1/2},$$

as well as 95% confidence intervals based on the central limit theorem $(\hat{\beta}_j - 1.96\hat{SD}_j, \hat{\beta}_j + 1.96\hat{SD}_j)$ ([4]). However, according to Lockhart et al. [7], con-

fidence intervals obtained by the bootstrap estimates are not optimal for the lasso.

References

[1] Hirotogu Akaike. *Selected Papers of Hirotugu Akaike*, chapter Information Theory and an Extension of the Maximum Likelihood Principle, pages 199–213. Springer New York, New York, NY, 1998.

[2] Marta Avalos, Yves Grandvalet, Nuria Duran Adroher, Ludivine Orriols, and Emmanuel Lagarde. Analysis of multiple exposures in the case-crossover design via sparse conditional likelihood. *Stat Med*, 2012.

[3] Marta Avalos, Ludivine Orriols, Helene Pouyes, Yves Grandvalet, Frantz Thiessard, and Emmanuel Lagarde. Variable selection on large case-crossover data: application to a registry-based study of prescription drugs and road traffic crashes. *Pharmacoepidemiol Drug Saf*, 2013.

[4] B. Efron and R.J. Tibshirani. *An Introduction to the Bootstrap*. Chapman & Hall/CRC Monographs on Statistics & Applied Probability. Taylor & Francis, 1994.

[5] Jerome H. Friedman, Trevor Hastie, and Rob Tibshirani. Regularization paths for generalized linear models via coordinate descent. *Journal of Statistical Software*, 33(1):1–22, 2 2010.

[6] Holly Janes, Lianne Sheppard, and Thomas Lumley. Overlap bias in the case-crossover design, with application to air pollution exposures. *Statistics in Medicine*, 24(2):285–300, 2005.

[7] Richard Lockhart, Jonathan Taylor, Ryan J. Tibshirani, and Robert Tibshirani. A significance test for the lasso. 2013.

[8] Thomas Lumley and Drew Levy. Bias in the case – crossover design: implications for studies of air pollution. *Environmetrics*, 11(6):689–704, 2000.

[9] J.M. Marin and C. Robert. *Bayesian Core: A Practical Approach to Computational Bayesian Statistics*. Springer Texts in Statistics. Springer, 2007.

[10] G. Rodríguez. Lecture notes on generalized linear models. 2007.

[11] Robert Tibshirani. Regression shrinkage and selection via the lasso. *Journal of the Royal Statistical Society, Series B*, 58:267–288, 1994.
