# Supplementary material for: Assessing short-term risk of ischemic stroke in relation to all prescribed medications
Source: Sci Rep. 2021 Nov 4;11:21673. doi: 10.1038/s41598-021-01115-7 (PMC8568938; doi:10.1038/s41598-021-01115-7)
Supplement: Supplementary file 2 — Supplementary Tables. [file 41598_2021_1115_MOESM2_ESM.docx]

| **TABLE S1. Odds ratio for ischemic stroke within 14 days after the drug was dispensed. BOLASSO results for drugs selected in NORWAY** | | | | | |
| --- | --- | --- | --- | --- | --- |
| **ATC** | **Generic name** | **Therapeutic Group** | **Exposed only in**  **Case period** | **Exposed**  **only in**  **Control period** | **BOLASSO  ESTIMATES**  **OR (95% CI)** |
| **A02BX13** | alginic acid | anti-ulcer agents | 0 | 7 | 0.18 (0.04-0.90) |
| **R03CC03** | terbutaline | adrenergic, systemic sue | 4 | 15 | 0.34 (0.10-1.13) |
| **R03DA04** | theophylline | systemic drug for obstructive airway disease | 33 | 62 | 0.41 (0.23-0.72) |
| **A11CC04** | calcitrol | Vitamin | 34 | 47 | 0.47 (0.22-1.02) |
| **N05CD03** | flunitrazepam | hypnotics and sedatives | 58 | 84 | 0.47 (0.29-0.76) |
| **H01BA02** | desmopressin | vasopressin and analogues | 13 | 26 | 0.48 (0.21-1.10) |
| **C09AA01** | captopril | ACE inhibitors | 24 | 44 | 0.49 (0.26-0.90) |
| **G03FA01** | norethisterone and estrogen | sex hormones and modulators | 33 | 56 | 0.56 (0.34-0.94) |
| **N06DX01** | memantine | anti-dementia drug | 55 | 70 | 0.62 (0.34-1.12) |
| **J07BB02** | infleunza, inactivated, split virus or surface antigen | Vaccine | 18 | 31 | 0.62 (0.35-1.12) |
| **R06AX27** | desloratadine | antihistamines | 56 | 73 | 0.70 (0.45-1.09) |
| **D07AC01** | betamethasone | corticosteriods, dermatologicals | 70 | 101 | 0.71 (0.50-1.01) |
| **N06AX16** | venlafaxine | antidepressants | 131 | 160 | 0.72 (0.52-1.00) |
| **J01XX05** | methenamine | antibiotics | 320 | 383 | 0.72 (0.56-0.93) |
| **S01AA01** | choramphenicol | antibiotics, opthalmologicals | 166 | 222 | 0.78 (0.63-0.96) |
| **R06AX13** | loratadine | antihistamines | 91 | 106 | 0.80 (0.57-1.13) |
| **R03AK06** | salmeterol and fluticasone | adrenergics, inhalants | 224 | 270 | 0.81 (0.66-1.00) |
| **S01ED51** | timolol, combinations | antiglaucoma in combination with beta-blocking agent | 253 | 300 | 0.83 (0.69-1.01) |
| **G04CB01** | finasteride | anti-benign prostatic hypertrophy drug | 113 | 137 | 0.84 (0.63-1.13) |
| **C09AA03** | lisinopril | ACE inhibitors | 244 | 273 | 0.84 (0.67-1.06) |
| **C03EA01** | hydrochorothiazide and potassium-sparing agents | diuretica | 179 | 204 | 0.86 (0.68-1.10) |
| **A02BA02** | ranitidine | anti-ulcer agents | 183 | 216 | 0.87 (0.69-1.10) |
| **M05BA04** | alendronic acid | bisphosphonates | 584 | 657 | 0.87 (0.73-1.03) |
| **C01DA14** | isosorbide mononitrate | vasodilators | 801 | 875 | 0.87 (0.74-1.02) |
| **A11EA** | vitamin B-complex, plain | Vitamin | 845 | 947 | 0.87 (0.74-1.03) |
| **C08DA01** | verapamil | calcium chain blocker | 266 | 276 | 0.88 (0.70-1.11) |
| **A02BC05** | esomeprazole | anti-peptic ulcer drug | 690 | 727 | 0.88 (0.76-1.02) |
| **A10BA02** | metformin | anti-diabetic drug | 887 | 985 | 0.88 (0.77-1.01) |
| **M01AE01** | ibuprofen | Anti-inflammatory drug | 274 | 279 | 0.89 (0.74-1.07) |
| **C09AA02** | enalapril | ACE inhibitors | 501 | 536 | 0.91 (0.78-1.07) |
| **C09DA01** | losartan and diuretics | angiotensin II receptor blocker | 363 | 399 | 0.92 (0.78-1.09) |
| **N05CF01** | zopiclone | hypnotics and sedatives | 2266 | 2471 | 0.92 (0.84-0.99) |
| **C10AA05** | atorvastatin | lipid modifying agent | 620 | 631 | 0.93 (0.81-1.07) |
| **C08CA01** | amlodipine | calcium chain blocker | 981 | 1038 | 0.93 (0.82-1.04) |
| **H03AA01** | levothyroxine sodium | anti-thyroid drug | 1058 | 1111 | 0.94 (0.83-1.05) |
| **A02BC02** | pantoprazole | anti-peptic ulcer drug | 863 | 883 | 0.96 (0.84-1.10) |
| **C10AA01** | simvastatin | lipid modifying agent | 2364 | 2401 | 0.99 (0.92-1.07) |
| **N02AX02** | tramadol | Opioids | 631 | 576 | 1,13 (0.97-1.31) |
| **B01AA03** | warfarin | antithrombotics | 761 | 793 | 1.00 (0.90-1.11) |
| **C07AB02** | metoprolol | beta blocking agent | 3584 | 3595 | 1.02 (0.95-1.10) |
| **C09CA06** | candesartan | Angiotensin II receptor blocker | 563 | 544 | 1.05 (0.90-1.23) |
| **C03CA01** | furosemide | Diuretica | 1815 | 1811 | 1.05 (0.95-1.16) |
| **A10BB12** | glimepiride | antidiabetic drug | 471 | 492 | 1.06 (0.90-1.25) |
| **M01AB05** | diclofenac | antiinflammatory drug | 454 | 443 | 1.06 (0.91-1.23) |
| **A12AX** | calcium, combinations with vitamin D and/or other drugs | Mineral Supplement | 867 | 871 | 1.06 (0.92-1.23) |
| **N05BA04** | oxazepam | anxiolytics | 995 | 979 | 1.06 (0.93-1.19) |
| **N02BE01** | paracetamol | analgesics | 2048 | 1959 | 1.06 (0.97-1.16) |
| **C09CA01** | losartan | Angiotensin II receptor blocker | 416 | 399 | 1.08 (0.91-1.28) |
| **N05BA01** | diazepam | anxiolytics | 798 | 705 | 1.09 (0.95-1.25) |
| **R03AC02** | salbutamol | adrenergics, inhalants | 445 | 420 | 1.11 (0.94-1.32) |
| **N06AB10** | escitalopram | antidepressants | 615 | 606 | 1.12 (0.95-1.32) |
| **C03CA02** | bumetanide | diuretica | 975 | 950 | 1.12 (0.97-1.30) |
| **C09AA05** | ramipril | ACE inhibitors | 899 | 864 | 1.14 (0.99-1.30) |
| **B01AC06** | acetylsalicyclic acid | antithrombotics | 4461 | 4411 | 1.14 (1.07-1.21) |
| **N06AX11** | mirtazapine | antidepressants | 294 | 284 | 1.17 (0.93-1.48) |
| **N02AA05** | oxycdone | Opioids | 312 | 261 | 1.18 (0.92-1.51) |
| **H02AB06** | prednisolone | glucorticosteriods | 924 | 817 | 1.22 (1.06-1.39) |
| **J01CF01** | dicloxacillin | antibiotics | 157 | 131 | 1.23 (0.96-1.58) |
| **B03AA07** | ferrous sulfate | anti-anemic drug | 353 | 335 | 1.24 (0.94-1.63) |
| **B01AC04** | clopidogrel | antithrombotics | 328 | 292 | 1.24 (1.01-1.52) |
| **N02AA59** | codeine, combinations excl. psycholeptics | Opioids | 1503 | 1288 | 1.24 (1.12-1.37) |
| **J01MA02** | ciprofloxacin | antibiotics | 216 | 154 | 1.26 (0.99-1.60) |
| **N03AX12** | gabapentin | antiepileptics | 189 | 151 | 1.27 (0.94-1.71) |
| **C03AB01** | bendroflumethiazide and potassium | Diuretica | 197 | 183 | 1.27 (0.98-1.64) |
| **G04CA02** | tamsulosin | alfa-adrenoreceptor antagonists, drugs used in benign prostatic hypertrophy | 339 | 308 | 1.27 (1.04-1.55) |
| **J01AA02** | doxycycline | antibiotics | 237 | 179 | 1.29 (1.04-1.60) |
| **J01EA01** | trimethoprim | antibiotics | 213 | 162 | 1.30 (1.04-1.63) |
| **B01AC07** | dipyridamole | antithrombotics | 500 | 446 | 1.33 (1.13-1.57) |
| **C09DB01** | valsartan and amlodipine | angiotensin II receptor blocker | 85 | 67 | 1.35 (0.93-1.95) |
| **J01FA01** | erythromycin | antibiotics | 122 | 82 | 1.37 (1.01-1.85) |
| **A07AA02** | oral nystatin | anti-fungal | 104 | 64 | 1.38 (0.95-2.01) |
| **J01CE02** | phenoxymethylpenicillin | antibiotics | 380 | 287 | 1.40 (1.19-1.65) |
| **R03AC13** | formoterol | adrenergics, inhalants | 58 | 42 | 1.41 (0.90-2.20) |
| **J01CA08** | pivmecillinam | antibiotics | 484 | 372 | 1.41 (1.21-1.64) |
| **N03AX16** | pregabalin | antiepileptics | 191 | 157 | 1.42 (1.02-1.97) |
| **A04AA01** | ondansetron | antiemetics and antinauseants | 100 | 57 | 1.44 (0.92-2.26) |
| **J01DB01** | cefalexin | antibiotics | 50 | 32 | 1.46 (0.92-2.32) |
| **M01AC06** | meloxicam | anti-inflammatory drug | 69 | 56 | 1.49 (0.97-2.29) |
| **B01AB04** | dalteparin | antithrombotics | 147 | 111 | 1.49 (1.05-2.10) |
| **C01AA05** | digoxin | cardiac glycosides | 141 | 123 | 1.52 (0.99-2.32) |
| **J01EE01** | sulfamethoxazole and trimethoprim | antibiotics | 121 | 83 | 1.52 (1.12-2.06) |
| **M02AA10** | ketoprofen | antiinflammatory drug | 62 | 43 | 1.53 (0.96-2.42) |
| **M01AB55** | diclofenac, combinations | antiinflammatory drug | 73 | 52 | 1.53 (0.97-2.42) |
| **C01BD01** | amiodarone | antiarrhythmics | 74 | 55 | 1.53 (0.98-2.40) |
| **N05AD01** | haloperidol | antipsychotics | 91 | 72 | 1.55 (0.95-2.53) |
| **B01AE07** | dabigatran etexilate | antithrombotics | 48 | 36 | 1.56 (0.90-2.70) |
| **J01CA04** | amoxicillin | antibiotics | 268 | 172 | 1.66 (1.32-2.09) |
| **N03AX14** | levetiracetam | antiepileptics | 61 | 45 | 1.69 (0.94-3.04) |
| **N06AA06** | trimipramine | antidepressants | 65 | 45 | 1.72 (0.99-3.00) |
| **B01AB05** | enoxaparin | antithrombotics | 124 | 73 | 1.73 (1.17-2.56) |
| **N02CC01** | sumatriptan | antimigraine drug | 48 | 33 | 1.76 (1.00-3.09) |
| **A03FA01** | metoclopramide | anti-emetic and propulsives | 417 | 243 | 1.81 (1.48-2.22) |
| **N02AB03** | fentanyl | Opioids | 189 | 96 | 1.87 (1.35-2.60) |
| **N05AB04** | prochlorperazine | antipsychotics | 107 | 71 | 1.92 (1.34-2.75) |
| **L04AA06** | myophenolic acid | immunosuppressants | 24 | 12 | 1.94 (0.85-4.39) |
| **B01AC30** | combinations | antithrombotics | 244 | 169 | 1.96 (1.54-2.50) |
| **G03XC01** | raloxifene | sex hormones and modulators | 25 | 13 | 2.58 (1.03-6.47) |
| **H02AB08** | triamcinolone | glucorticosteriods | 15 | 3 | 3.19 (1.01-10.12) |
| **S01EC01** | acetazolamide | antiglaucoma drug | 14 | 7 | 3.36 (0.99-11.41) |
| **A04AA03** | tropisetron | antiemetics and antinauseants | 10 | 2 | 4.28 (1.26-14.60) |
| **N02AA01** | morphine | Opioids | 165 | 77 | 4.53 (2.67-7.68) |
| **N02AG01** | morphine and antispasmodics | Opioids | 46 | 0 | 33,99 (15.56-74.26) |

[All generic names listed according to the Anatomical Therapeutic Chemical (ATC), 5^th^ level

Case crossover analysis, case period (1-14 days) and control period (29-42 days) before the index-date for the diagnosis of ischemic stroke]

| **Table S2. Odds ratio for ischemic stroke within 14 days after the prescribed medication was dispensed. BOLASSO results for drugs selected in SWEDEN** | | | | | |
| --- | --- | --- | --- | --- | --- |
| **ATC** | **Generic name** | **Therapeutic Group** | **Exposed**  **only in**  **Case**  **period** | **Exposed**  **only in**  **Control period** | **Bolasso**  **OR (95% CI)** |
| N02AG01 | morphine and antispasmodics | Opioids | 121 | 35 | 4.24 (2.71-6.64) |
| A03AB02 | glycopyrronium bromide | anticholinergics, drugs for gastrointestinal disorders | 20 | 5 | 3.21 (1.15-9.01) |
| N02AG02 | ketobemidone and antispasmodics | Opioids | 31 | 15 | 2.77 (1.02-7.50) |
| R03DA02 | choline theophyllinate | Systemic drugs for obstructive airway disease | 30 | 19 | 2.34 (0.98-5.56) |
| N01BB20 | Amides combinations | local anesthetics | 60 | 31 | 2.31 (1.29-4.12) |
| R06AA02 | diphenhydramine | antihistamines | 22 | 9 | 2.30 (0.98-5.41) |
| B01AF02 | apixaban | antithrombotics | 33 | 17 | 2.04 (1.04-4.00) |
| C09CA07 | telmisartan | angiotension II Receptor Blockers | 40 | 22 | 1.88 (1.07-3.33) |
| R06AE05 | meclozine | antihistamines | 93 | 51 | 1.79 (1.21-2.65) |
| B01AF01 | rivaroxaban | antithrombotics | 39 | 23 | 1.76 (0.93-3.35) |
| J01DB05 | cefadroxil | antibiotics | 182 | 112 | 1.71 (1.31-2.23) |
| B02AA02 | tranexamic acid | antihemorrhagics | 61 | 43 | 1,70 (1.06-2.73) |
| J01MA06 | norfloxacin | antibiotics | 103 | 65 | 1.61 (1.17-2.21) |
| A07AA02 | nystatin | antifungal | 270 | 170 | 1.57 (1.28-1.94) |
| N02CC01 | sumatriptan | antimigraine drug | 105 | 80 | 1.57 (1.07-2.31) |
| B01AC24 | ticagrelor | antithrombotics | 78 | 47 | 1.57 (1.01-2.44) |
| J01MA02 | ciprofloxacin | antibiotics | 826 | 547 | 1.52 (1.34-1.72) |
| J01EA01 | trimethoprim | antibiotics | 446 | 309 | 1.52 (1.29-1.78) |
| N02AA01 | morphine | opioids | 1316 | 1048 | 1.51 (1.30-1.76) |
| P01AB01 | metronidazole | antiprotozoals | 230 | 138 | 1.46 (1.15-1.86) |
| J01AA02 | doxycycline | antibiotics | 695 | 459 | 1.46 (1.28-1.66) |
| J01CA04 | amoxicillin | antibiotics | 475 | 317 | 1.46 (1.25-1.71) |
| B01AC07 | dipyridamole | antithrombotics | 410 | 298 | 1.44 (1.22-1.70) |
| B01AC04 | clopidogrel | antithrombotics | 1265 | 1018 | 1.40 (1.25-1.57) |
| B01AB05 | enoxaparin | antithrombotics | 126 | 101 | 1.39 (0.97-1.98) |
| A03FA01 | metoclopramide | anti-emetic and propulsives | 549 | 397 | 1.38 (1.17-1.64) |
| N05CM02 | clomethiazole | Hypnotics and sedatives | 511 | 478 | 1.33 (1.04-1.70) |
| R05FA02 | opium derivatives and expectorants | cough suppressants and expectorants, combinations | 665 | 474 | 1.32 (1.16-1.50) |
| C07FB02 | metoprolol and felodipine | beta blocking agent and calcium channel blockers | 114 | 94 | 1.31 (0.95-1.81) |
| N02AB03 | fentanyl | opioids | 392 | 318 | 1.31 (1.07-1.61) |
| B01AB04 | dalteparin | antithrombotics | 597 | 459 | 1.30 (1.10-1.53) |
| B03AA01 | ferrous glycine sulfate | antianemic preparations | 171 | 163 | 1.28 (0.94-1.73) |
| H02AB01 | betamethasone | glucocorticoids | 525 | 392 | 1.23 (1.05-1.43) |
| J01CA08 | pivmecillinam | antibiotics | 700 | 588 | 1.22 (1.09-1.37) |
| J01CE02 | phenoxymethylpenicillin | antibiotics | 744 | 605 | 1.22 (1.08-1.37) |
| A06AB08 | sodium picosulfate | anticonstipation drug | 582 | 444 | 1.21 (1.05-1.38) |
| R05CB10 | combinations | expectorants, excl. combinations with cough suppressants | 480 | 400 | 1.16 (1.00-1.35) |
| C07AB07 | bisoprolol | beta blocking agents | 3977 | 3768 | 1.14 (1.06-1.23) |
| C01DA02 | glyceryl trinitrate | vasodilator | 1724 | 1569 | 1.14 (1.05-1.23) |
| N02AA05 | oxycodone | Opioids | 2066 | 1963 | 1.14 (1.01-1.27) |
| C09DA01 | losartan and diuretics | Angiotensin II Receptor Blockers, combn. | 647 | 627 | 1.10 (0.96-1.26) |
| N06AX11 | mirtazapine | antidepressants | 3018 | 2998 | 1.09 (0.97-1.23) |
| A12BA01 | potassium chloride | Mineral supplements | 2369 | 2288 | 1.07 (0.96-1.20) |
| C03DA01 | spironolactone | diuretica | 2840 | 2803 | 1.06 (0.97-1.17) |
| M01AB05 | diclofenac | anti-inflammatory drug | 1191 | 1141 | 1.05 (0.96-1.15) |
| C09AA02 | enalapril | ACE inhibitors | 6740 | 6716 | 1.04 (0.99-1.10) |
| B01AA03 | warfarin | antithrombotics | 1649 | 1612 | 1.04 (0.97-1.12) |
| C08CA01 | amlodipine | calcium chain blockers | 3058 | 3070 | 1.03 (0.96-1.11) |
| C07AB02 | metoprolol | beta blocking agents | 11540 | 11596 | 1.02 (0.97-1.06) |
| B01AC06 | acetylsalicyclic acid | antithrombotics | 18467 | 18667 | 1.01 (0.98-1.05) |
| A02BC01 | omeprazole | Anti-peptic ulcer drug | 8333 | 8385 | 1.00 (0.95-1.06) |
| C07AB03 | atenolol | beta blocking agents | 3470 | 3582 | 0.99 (0.93-1.06) |
| C03CA01 | furosemide | diuretics | 13839 | 14075 | 0.99 (0.94-1.04) |
| C09AA05 | ramipril | ACE inhibitors | 2576 | 2568 | 0.98 (0.90-1.07) |
| N05BA04 | oxazepam | anxiolytics | 4353 | 4474 | 0.98 (0.91-1.06) |
| C08CA02 | felodipine | calcium chain blockers | 3957 | 4115 | 0.97 (0.91-1.04) |
| N02BE01 | paracetamol | analgesics | 13481 | 13744 | 0.97 (0.93-1.01) |
| A10BA02 | metformin | antidiabetic drug | 3062 | 3247 | 0.96 (0.89-1.04) |
| C10AA01 | simvastatin | lipid modifying agents | 6672 | 6871 | 0.96 (0.91-1.01) |
| H03AA01 | levothyroxine sodium | anti-thyroid agent | 5675 | 5831 | 0.95 (0.89-1.02) |
| A10AD05 | insulin aspart | anti-diabetic drug | 977 | 1048 | 0.95 (0.86-1.05) |
| C09CA01 | losartan | ACE inhibitors | 2047 | 2133 | 0.94 (0.86-1.03) |
| B03BA01 | cyanocobalamin | vasolidators | 8369 | 8699 | 0.94 (0.88-1.00) |
| R03BB04 | tiotropium bromide | Anticholinergics, drug for obstructive airways diseases | 723 | 773 | 0.94 (0.83-1.06) |
| C01DA14 | isosorbide monnitrate | vasodilators | 4469 | 4632 | 0.93 (0.86-1.01) |
| S01ED51 | timolol, combinations | antiglaucoma drug | 751 | 833 | 0.93 (0.83-1.04) |
| S01EE01 | latanoprost | antiglaucoma drug | 808 | 928 | 0.91 (0.82-1.02) |
| A11EA | vitamin B-complex, plain | Vitamins | 1941 | 2052 | 0.90 (0.80-1.02) |
| N05CF02 | zolpidem | Hypnotics and sedatives | 2857 | 3033 | 0.90 (0.83-0.98) |
| D07AC01 | betamethasone | corticosteriods, dermatologicals | 394 | 454 | 0.90 (0.78-1.05) |
| A12AX | Calcium, combinations with vitamin D and/or other drugs | Mineral supplements | 4057 | 4319 | 0.90 (0.84-0.97) |
| G04CB01 | finasteride | testosterone-5-alpha reductase inhibitors, use for benign prostatic hypertrophy treatment | 517 | 587 | 0.90 (0.80-1.02) |
| N05CF01 | zopiclone | Hypnotics and sedatives | 6046 | 6312 | 0.90 (0.85-0.96) |
| M04AA01 | allopurinol | antigout agents | 1810 | 1920 | 0.90 (0.81-1.00) |
| A10BB01 | glibenclamide | antidiabetic drug | 1015 | 1118 | 0.89 (0.78-1.02) |
| N06AB04 | citalopram | antidepressants | 5012 | 5216 | 0.89 (0.82-0.97) |
| C09CA06 | candesartan | Angiotension II Receptor blockers | 1735 | 1837 | 0.89 (0.81-0.98) |
| G03CA04 | estriol | Estrogens | 1092 | 1166 | 0.89 (0.77-1.03) |
| D02AE01 | carbamide | Emollient and protectives | 964 | 1088 | 0.89 (0.80-0.98) |
| S01XA20 | artificial tears and other indifferent preparations | Opthalmologicals | 879 | 1006 | 0.87 (0.78-0.97) |
| G04BD07 | tolterodine | antispasmodics used in urogenital tractus | 631 | 703 | 0.86 (0.72-1.01) |
| A03AX13 | silicones | functional gastrointestinal disorder treatment | 463 | 517 | 0.85 (0.69-1.03) |
| G03CA03 | estradiol | Estrogens | 789 | 933 | 0.84 (0.76-0.94) |
| N06DA02 | donepezil | antidementia drugs | 999 | 1050 | 0.84 (0.69-1.03) |
| J01XX05 | methenamine | antibiotics | 578 | 627 | 0.83 (0.67-1.04) |
| A01AA01 | sodium fluoride | Caries prophylactic agents | 505 | 599 | 0.83 (0.72-0.94) |
| A11CC03 | alfacalcidol | Vitamins A and D, combination | 311 | 342 | 0.81 (0.63-1.05) |
| N05CD02 | nitrazepam | Hypnotics and sedatives | 377 | 416 | 0.80 (0.63-1.01) |
| A02BC03 | lansoprazole | anti-peptic ulcer drugs | 258 | 324 | 0.80 (0.65-0.98) |
| S01AA01 | choramphenicol | antibiotics, opthalmologicals | 194 | 242 | 0.79 (0.63-0.99) |
| D07AD01 | clobetasol | corticosteriods, dermatological prepartions | 150 | 209 | 0.76 (0.60-0.97) |
| D07BC01 | betamethasone and antiseptics | corticosteriods, dermatological prepartions | 57 | 80 | 0.73 (0.50-1.07) |
| G04BE03 | sildenafil | antierectile dysfunction drug | 166 | 225 | 0.72 (0.57-0.91) |
| A10BB12 | glimepiride | antidiabetic drug | 186 | 230 | 0.72 (0.55-0.95) |
| S01EB01 | pilocarpine | antiglaucoma drug | 81 | 109 | 0.72 (0.51-1.01) |
| G04BE01 | alprostadil | antierectile dysfunction drug | 56 | 84 | 0.72 (0.48-1.07) |
| S01BC10 | nepafenac | antiinflammatory agents | 66 | 89 | 0.70 (0.46-1.05) |
| G04CB02 | dutasteride | anti-benign prostatic hypertrophy drug | 54 | 77 | 0.69 (0.47-1.00) |
| N06AA04 | clomipramine | antidepressants | 178 | 221 | 0.69 (0.48-0.98) |
| N06DA04 | galantamine | antidementia drug | 356 | 419 | 0.65 (0.47-0.90) |
| N05AA02 | levomepromazine | antipsychotics | 269 | 307 | 0.62 (0.42-0.92) |
| C10AB04 | gemfibrozil | Lipid modifying agents | 40 | 59 | 0.61 (0.36-1.06) |
| G04BE08 | tadalafil | Anti-erectile dysfunction drug | 86 | 136 | 0.61 (0.44-0.84) |
| N06AB05 | paroxetine | antidepressants | 228 | 288 | 0.61 (0.45-0.82) |
| N05AN01 | lithium | antipsychotics | 239 | 281 | 0.59 (0.39-0.89) |
| N05AF01 | flupentixol | antipsychotics | 95 | 120 | 0.52 (0.31-0.86) |
| D07XA01 | hydrocortisone | corticosteriods, dermatological prepartions | 14 | 34 | 0.50 (0.23-1.05) |
| N05BE01 | buspirone | anxiolytics | 61 | 77 | 0.43 (0.20-0.93) |
| B03AA02 | ferrous fumarate | antianemic preparations | 65 | 95 | 0.39 (0.20-0.74) |
| L01BC02 | fluorouracil | antineoplastic agents | 4 | 16 | 0.36 (0.14-0.96) |
| L03AA02 | filgrastim | immunostimulants | 4 | 12 | 0.21 (0.06-0.67) |

[All generic names listed according to the Anatomical Therapeutic Chemical (ATC), 5^th^ level

Case crossover analysis, case period (1-14 days) and control period (29-42 days) before the index-date for the diagnosis of ischemic stroke]

| **TABLE S3. Odds ratio for ischemic stroke within 30 days after the drug was dispensed. BOLASSO results for drugs selected in NORWAY** | | | | | |
| --- | --- | --- | --- | --- | --- |
| **ATC** | **Generic name** | **Therapeutic Group** | **Exposed**  **only in**  **Case**  **period** | **Exposed**  **only in**  **Control period** | **Bolasso**  **OR (95% CI)** |
| D01AE15 | terbinafine | antifungals | 20 | 45 | 0.49 (0.27-0.89) |
| S01BC10 | nepafenac | antiinflammatory agents | 28 | 51 | 0.61 (0.35-1.06) |
| S01GX01 | cromoglicic acid | antiallergics, opthalmologicals | 30 | 44 | 0.62 (0.35-1.12) |
| A12BA02 | potassium citrate | mineral supplements | 48 | 62 | 0.64 (0.37-1.10) |
| S01EC03 | dorzolamide | antiglaucoma drug | 42 | 58 | 0.65 (0.40-1.06) |
| C09AA01 | captopril | ACE inhibitors | 67 | 94 | 0.67 (0.44-1.03) |
| R01AD08 | fluticasone | corticosteroids, decongestants and other nasal preparations | 44 | 62 | 0.68 (0.43-1.03) |
| D05AX52 | calcipotriol, combinations | antipsoriatics | 51 | 72 | 0.68 (0.45-1.03) |
| S01BA01 | dexamethasone | antiinflammatory agents | 102 | 135 | 0.71 (0.50-1.00) |
| N05AA02 | levomepromazine | antipsychotics | 248 | 266 | 0.74 (0.54-1.00) |
| A10AD05 | insulin aspart | antidiabetic drug | 176 | 210 | 0.76 (0.58-1.00) |
| N06AX16 | venlafaxine | antidepressants | 285 | 314 | 0.77 (0.59-1.02) |
| D07AD01 | clobetasol | corticosteriods, dermatological prepartions | 140 | 176 | 0.79 (0.61-1.02) |
| R01AD09 | mometasone | corticosteriods, decongestants and other nasal preparations | 213 | 243 | 0.79 (0.62-1.00) |
| D07AB02 | hydrocortisone butyrate | corticosteriods | 129 | 146 | 0.80 (0.62-1.04) |
| S01AA13 | fusidic acid | antibiotics | 92 | 115 | 0.82 (0.61-1.09) |
| C01AA04 | digitoxin | cardiac glycosides | 1189 | 1255 | 0.82 (0.71-0.96) |
| N06AB04 | citalopram | antidepressants | 546 | 553 | 0.84 (0.67-1.04) |
| C09DA01 | losartan and diuretics | angiotension II receptor blockers | 782 | 842 | 0.85 (0.74-0.97) |
| C09BA02 | enalapril and diuretics | ACE inhibitors, combinations | 278 | 309 | 0.85 (0.69-1.05) |
| C09AA03 | lisinopril | ACE inhibitors | 534 | 569 | 0.88 (0.73-1.05) |
| A02BA02 | ranitidine | antipeptic ulcer drugs | 401 | 426 | 0.88 (0.73-1.07) |
| S01EE01 | latanoprost | antiglaucoma drug | 531 | 566 | 0.89 (0.76-1.04) |
| C01DA02 | glyceryl trinitrate | vasodilators | 671 | 714 | 0.90 (0.79-1.01) |
| C09CA06 | candesartan | angiotension II receptor blockers | 1149 | 1164 | 0.90 (0.80-1.02) |
| C09DA06 | candesartan and diuretics | angiotension II receptor blockers, combinations | 546 | 569 | 0.90 (0.78-1.05) |
| A10BA02 | metformin | anti-diabetic drug | 1896 | 2031 | 0.91 (0.83-1.01) |
| A02BC05 | esomeprazole | anti-peptic ulcer drugs | 1480 | 1493 | 0.93 (0.83-1.05) |
| A10BB12 | glimepiride | anti-diabetic drug | 981 | 1038 | 0.93 (0.82-1.07) |
| A11EA | vitamin B-complex, plain | vitamins | 1837 | 1869 | 0.94 (0.83-1.06) |
| C01DA14 | isosorbide mononitrate | vasodilators | 1725 | 1708 | 0.95 (0.84-1.06) |
| C10AA01 | simvastatin | lipid modifying agents | 4911 | 4882 | 0.96 (0.91-1.02) |
| C08CA01 | amlodipine | calcium chain blockers | 2048 | 2051 | 0.97 (0.90-1.06) |
| B01AA03 | warfarin | antithrombotics | 1546 | 1606 | 0.99 (0.91-1.08) |
| N05CF01 | zopiclone | hypnotics and sedatives | 4833 | 4770 | 1.01 (0.95-1.08) |
| A12AX | Calcium, combinations with vitamin D and/or other drugs | Mineral supplements | 1849 | 1799 | 1.06 (0.94-1.19) |
| C07AB02 | metoprolol | beta blocking agents | 7389 | 7048 | 1.08 (1.02-1.14) |
| N02BE01 | paracetamol | analgesics | 4112 | 3681 | 1.09 (1.01-1.17) |
| A02BC02 | pantoprazole | anti-peptic ulcer drugs | 1766 | 1615 | 1.10 (0.98-1.24) |
| C03CA01 | furosemide | Diuretica | 3742 | 3638 | 1.10 (1.02-1.20) |
| R05CB01 | acetylcysteine | Expectorants, excl. combinations with cough suppressants | 940 | 834 | 1.11 (0.98-1.25) |
| B01AC06 | acetylsalicyclic acid | antithrombotics | 9116 | 8712 | 1.11 (1.06-1.16) |
| N06AB10 | escitalopram | antidepressants | 1259 | 1165 | 1.13 (0.98-1.29) |
| S01XA20 | artificial tears and other indifferent preparations | opthalmologicals | 290 | 278 | 1.13 (0.91-1.40) |
| R05DA01 | ethylmorphine | expectorants, excl. combinations with cough suppressants | 511 | 423 | 1.13 (0.98-1.31) |
| G04CA02 | tamsulosin | alfa-adrenoreceptor antagonists, used in benign prostatic hypertrophy | 696 | 646 | 1.14 (0.98-1.32) |
| N05BA01 | diazepam | anxiolytics | 1552 | 1386 | 1.15 (1.03-1.29) |
| M01AB05 | diclofenac | anti-inflammatory agent | 876 | 761 | 1.15 (1.02-1.30) |
| N05BA04 | oxazepam | anxiolytics | 2075 | 1933 | 1.16 (1.04-1.29) |
| C08CA05 | nifedipine | calcium channel blockers | 699 | 652 | 1.16 (0.99-1.36) |
| N02AE01 | buprenorphine | opioids | 406 | 358 | 1.17 (0.93-1.47) |
| N02AA59 | codeine, combinatiosn excl. psycholeptics | opioids | 2945 | 2624 | 1.17 (1.08-1.28) |
| C03CA02 | bumetanide | diuretics | 1970 | 1813 | 1.19 (1.05-1.35) |
| N05BB01 | hydroxyzine | anxiolytics | 333 | 330 | 1.20 (0.93-1.55) |
| B03BA03 | hydroxocobalamin | Vitamin B12 | 198 | 163 | 1.21 (0.97-1.50) |
| J01CA08 | pivmecillinam | antibiotics | 885 | 739 | 1.21 (1.09-1.35) |
| M01AE02 | naproxen | antiinflammatory | 219 | 196 | 1.21 (0.96-1.54) |
| B01AC07 | dipyridamole | antithrombotics | 994 | 916 | 1.22 (1.07-1.40) |
| H02AB06 | prednisolone | corticosteriods | 1811 | 1548 | 1.22 (1.10-1.35) |
| C03DA01 | spironolactone | diuretics | 657 | 575 | 1.23 (1.02-1.48) |
| J01XE01 | nitrofurantoin | antibiotics | 262 | 201 | 1.25 (0.99-1.57) |
| J01CE02 | phenoxymethylpenicillin | antibiotics | 688 | 548 | 1.25 (1.10-1.41) |
| J01CF01 | dicloxacillin | antibiotics | 302 | 248 | 1.25 (1.04-1.52) |
| N06AX11 | mirtazapine | antidepressants | 629 | 566 | 1.26 (1.02-1.55) |
| J01EE01 | sulfamethoxazole and trimethoprim | antibiotics | 202 | 157 | 1.31 (1.03-1.66) |
| J01MA02 | ciprofloxacin | antibiotics | 386 | 278 | 1.31 (1.09-1.58) |
| C05AA04 | prednisolone | corticosteroids used in anti- hemorrhoids and fissures treatment | 116 | 91 | 1.32 (0.96-1.82) |
| M01AH05 | etoricoxib | anti-inflammatory | 133 | 100 | 1.33 (0.98-1.80) |
| N02AA05 | oxycodone | Opioids | 604 | 474 | 1.35 (1.06-1.72) |
| N02AX02 | tramadol | Opioids | 1286 | 1026 | 1.35 (1.18-1.54) |
| J01AA02 | doxycycline | antibiotics | 472 | 341 | 1.36 (1.16-1.59) |
| A07AA02 | nystatin | antifungal | 177 | 118 | 1.37 (1.04-1.82) |
| J01CA04 | amoxicillin | antibiotics | 472 | 330 | 1.37 (1.17-1.62) |
| C01BD01 | amiodarone | antiarrhythmics | 126 | 109 | 1.39 (0.92-2.12) |
| A06AB02 | bisacodyl | anticonstipation drug | 170 | 147 | 1.43 (0.93-2.18) |
| B01AC04 | clopidogrel | antithrombotics | 660 | 535 | 1.43 (1.21-1.68) |
| B03AA07 | ferrous sulfate | antianemic drug | 732 | 664 | 1.43 (1.12-1.83) |
| B01AB05 | enoxaparin | antithrombotics | 224 | 141 | 1.46 (1.07-2.00) |
| J01FA01 | erythromycin | antibiotics | 206 | 129 | 1.50 (1.18-1.89) |
| A03FA01 | metoclopramide | anti-emetic and propulsives | 707 | 481 | 1.50 (1.27-1.77) |
| H02AB04 | methylprednisolone | glucorticosteriods | 129 | 74 | 1.50 (0.98-2.30) |
| N02CC01 | sumatriptan | antimigraine agents | 91 | 70 | 1.52 (0.94-2.46) |
| A06AD65 | macrogol, combinations | anticonstipation drug | 62 | 34 | 1.58 (0.98-2.55) |
| B01AB04 | dalteparin | antithrombotics | 261 | 173 | 1.70 (1.25-2.30) |
| J05AH02 | oseltamivir | antivirals | 26 | 14 | 1.87 (0.91-3.83) |
| S03CA01 | dexamethasone and antiinfectives | corticosteroids and antiinfectives in combination | 33 | 21 | 1.99 (0.99-3.99) |
| N05AB04 | prochlorperazine | antipsychotics | 198 | 132 | 2.00 (1.48-2.71) |
| N02AA55 | oxycodone and naloxone | Opioids | 53 | 31 | 2.18 (0.91-5.22) |
| M01AB01 | indometacin | antiinflammatory drug | 30 | 16 | 2.27 (1.03-5.01) |
| A11CC01 | ergocalciferol | vitamin D and analogues | 22 | 9 | 2.42 (0.93-6.30) |
| B01AC30 | Platelet aggregation inhibitors excl. heparin | antithrombotics | 439 | 283 | 2.42 (1.91-3.08) |
| B01AC24 | ticagrelor | antithrombotics | 39 | 19 | 2.49 (1.02-6.06) |
| G03XC01 | raloxifene | Sex hormones and modulators | 41 | 27 | 2.54 (0.93-6.94) |
| N02AB03 | fentanyl | Opioids | 319 | 162 | 2.62 (1.77-3.89) |
| N02AA01 | morphine | Opioids | 252 | 117 | 3.03 (2.04-4.52) |
| A10BX07 | liraglutide | antidiabetic drug | 23 | 13 | 3.10 (0.93-10.29) |
| B01AF02 | apixaban | antithrombotics | 26 | 13 | 3.15 (0.93-10.69) |
| N02AG02 | ketobemidone and antispasmodics | Opioids | 39 | 13 | 3.52 (1.29-9.62) |
| N05CM02 | clomethiazole | Hypnotics and sedatives | 88 | 46 | 4.13 (1.87-9.15) |
| N02AG01 | morphine and antispasmodics | Opioids | 49 | 1 | 15.70 (6.01-41.0) |

[All generic names listed according to the Anatomical Therapeutic Chemical (ATC), 5^th^ level

Case crossover analysis, case-period (1-30 days) and control period (61-90 days) before the index-date for the diagnosis of first ischemic stroke]

| **Table S4 Odd ratios for ischemic stroke within 30 days after the drug was dispensed. BOLASSO results for drugs selected in SWEDEN** | | | | | |
| --- | --- | --- | --- | --- | --- |
| **ATC** | **Generic name** | **Therapeutic Group** | **Exposed**  **only in**  **Case**  **period** | **Exposed**  **only in**  **Control period** | **Bolasso**  **OR (95% CI)** |
| M01AC05 | lornoxicam | anti-inflammatory agent | 10 | 0 | 0.22 (0.06-0.74) |
| A11CC04 | calcitrol | Vitamins | 11 | 23 | 0.26 (0.09-0.76) |
| N06BC01 | caffeine | psychoanaleptics (for ADHD treatment.) | 7 | 22 | 0.31 (0.10-0.96) |
| N07AA02 | pyridostigmine | parasympathomimetics | 46 | 65 | 0.49 (0.23-1.04) |
| G03FB05 | norethisterone & estrogen, sequential prep. | sex hormones and modulators | 18 | 35 | 0.55 (0.29-1.05) |
| N05AN01 | lithium | antipsychotics | 520 | 577 | 0.56 (0.39-0.79) |
| N05AB03 | perphenazine | antipsychotics | 160 | 182 | 0.59 (0.36-0.99) |
| C10AB04 | gemfibrozil | lipid modifying agent | 99 | 132 | 0.65 (0.44-0.95) |
| G04BE01 | alprostadil | erectile dysfunction treatment | 139 | 201 | 0.66 (0.48-0.91) |
| N05AA02 | levomepromazine | antipsychotics | 607 | 642 | 0.67 (0.48-0.93) |
| A10AB01 | insulin (human) | anti-diabetic drug | 72 | 96 | 0.68 (0.46-1.01) |
| C10AC01 | colestyramine | lipid modifying agent | 87 | 104 | 0.69 (0.451.04) |
| G03FA01 | norethisterone & estrogen, fixed | sex hormones and modulators | 141 | 196 | 0.69 (0.52-0.92) |
| S01GX01 | cromoglicic acid | anti-allergics, opthalmologicals | 143 | 192 | 0.73 (0.56-0.95) |
| C08CA03 | isradipine | calcium channel blockers | 195 | 233 | 0.73 (0.56-0.96) |
| G04CB02 | dutasteride | anti-benign prostatic hypertrophy drug | 120 | 151 | 0.75 (0.58-0.97) |
| A02BC03 | lansoprazole | anti-peptic ulcer drugs | 576 | 716 | 0.75 (0.64-0.89) |
| L02BA01 | tamoxifen | antiestrogens | 245 | 282 | 0.78 (0.58-1.04) |
| C08CA05 | nifedipine | calcium channel blockers | 350 | 398 | 0.78 (0.64-0.96) |
| N02AB01 | ketobemidone | opioids | 354 | 381 | 0.80 (0.62-1.03) |
| C10AX09 | ezetimibe | lipid modifying agents | 201 | 240 | 0.81 (0.65-1.00) |
| A10AD04 | insulin lispro | anti-diabetic drug | 264 | 300 | 0.81 (0.63-1.03) |
| C05AA04 | prednisolone | corticosteroids, anti-hemorrhoids agents | 236 | 272 | 0.81 (0.65-1.01) |
| R01AD05 | budesonide | corticosteroids, decongestants and other nasal preparations | 305 | 368 | 0.82 (0.68-0.98) |
| M05BA07 | risedronic acid | biphosphonates | 363 | 406 | 0.83 (0.68-1.02) |
| A10AE04 | insulin glargine | anti-diabetic drugs | 865 | 934 | 0.85 (0.75-0.96) |
| R06AE07 | cetirizine | antihistamines | 522 | 558 | 0.85 (0.71-1.01) |
| R03BB01 | ipratropium bromide | anticholinergics | 541 | 588 | 0.86 (0.73-1.02) |
| C07AA07 | sotalol | beta-blocking agents | 1038 | 1107 | 0.88 (0.77-1.01) |
| G04BE03 | sildenafil | erectile dysfunction treatment | 387 | 432 | 0.89 (0.75-1.04) |
| S03CA04 | hydrocortisone and antiinfectives | corticosteroids and antiinfectives in combination, Opthalmogicals and Otological | 695 | 764 | 0.89 (0.79-0.99) |
| A03AX13 | silicones | functional gastrointestional disorder treatment | 952 | 988 | 0.89 (0.75-1.05) |
| A10BB01 | glibenclamide | anti-diabetic drugs | 2237 | 2329 | 0.89 (0.81-0.99) |
| G03CA03 | estradiol | Estrogens | 1760 | 1869 | 0.90 (0.83-0.97) |
| S01EC04 | [brinzolamide](https://www.whocc.no/atc_ddd_index/?code=S01EC04) | anti-glaucoma agents | 536 | 568 | 0.90 (0.77-1.05) |
| M04AA01 | allopurinol | anti-gout agents | 3827 | 3894 | 0.90 (0.83-0.98) |
| M01AX05 | glucosamine | anti-inflammatory drug | 817 | 847 | 0.91 (0.80-1.04) |
| D01AC20 | imidazoles/triazoles in combination with corticosteroids | anti-fungals | 782 | 829 | 0.91 (0.82-1.02) |
| B01AA03 | warfarin | antithrombotics | 3323 | 3406 | 0.92 (0.86-0.97) |
| G03CA04 | estriol | Estrogens | 2350 | 2408 | 0.92 (0.82-1.02) |
| S01XA20 | artificial tears and other indifferent preparations | Opthalmologicals | 1958 | 2073 | 0.92 (0.85-1.00) |
| G04BD07 | tolterodine | antispasmodics used in urogenital tractus | 1379 | 1424 | 0.92 (0.81-1.05) |
| A01AA01 | sodium fluoride | caries prophylactic agent | 1124 | 1199 | 0.93 (0.84-1.02) |
| N05BB01 | hydroxyzine | anxiolytics | 2178 | 2228 | 0.93 (0.84-1.03) |
| M01AE02 | naproxen | anti-inflammatory agent | 916 | 996 | 0.93 (0.83-1.04) |
| S01EE01 | latanoprost | anti-glaucoma agent | 1753 | 1847 | 0.93 (0.86-1.01) |
| D02AE01 | carbamide | emollient and protectives, dermatologicals | 2113 | 2207 | 0.94 (0.87-1.01) |
| C08CA02 | felodipine | calcium chain blocker | 8411 | 8614 | 0.94 (0.89-0.99) |
| H03AA01 | Levothyroxine sodium | anti-thyroid drugs | 11882 | 11795 | 0.94 (0.89-0.99) |
| A10AC01 | insulin (human) | anti-diabetic agents | 1559 | 1647 | 0.94 (0.86-1.03) |
| C07AB03 | atenolol | beta-blocking agents | 7404 | 7568 | 0.95 (0.90-1.00) |
| C03EA01 | [hydrochlorothiazide and potassium-sparing agents](https://www.whocc.no/atc_ddd_index/?code=C03EA01) | diuretics | 2632 | 2722 | 0.95 (0.88-1.03) |
| A10BA02 | metformin | anti-diabetic drug | 6451 | 6635 | 0.95 (0.90-1.01) |
| N05CF02 | zolpidem | hypnotics and sedatives | 6072 | 6081 | 0.95 (0.90-1.02) |
| A12AX | calcium | calcium, combinations with vitamin D and/or other drugs | 8715 | 8703 | 0.96 (0.91-1.01) |
| C03AA01 | bendroflumethiazide | diuretics | 3629 | 3690 | 0.96 (0.90-1.03) |
| C01DA14 | isosorbide monnitrate | vasodilators | 9485 | 9368 | 0.97 (0.91-1.03) |
| C10AA01 | simvastatin | lipid modifying agents | 13949 | 13753 | 0.97 (0.94-1.01) |
| C08CA01 | amlodipine | calcium chain blockers | 6368 | 6261 | 1.01 (0.95-1.06) |
| B03BA01 | cyanocobalamin | vitamin B12 | 17757 | 17251 | 1.02 (0.98-1.07) |
| B01AC06 | acetylsalicyclic acid | anti-thrombotics | 38514 | 37348 | 1.03 (1.00-1.06) |
| S01ED51 | timolol, combinations | antiglaucoma agents | 1620 | 1619 | 1.03 (0.94-1.13) |
| C09AA05 | ramipril | ACE inhibitors | 5398 | 5152 | 1.03 (0.96-1.11) |
| C10AA05 | atorvastatin | lipid modifying agents | 2110 | 1998 | 1.04 (0.95-1.13) |
| N02AX02 | tramadol | opioids | 4062 | 3993 | 1.04 (0.97-1.11) |
| N02BE01 | paracetamol | analgesics | 28071 | 26895 | 1.04 (1.01-1.08) |
| A06AD65 | macrogol, combinations | anti-constipation drugs | 1879 | 1616 | 1.06 (0.98-1.14) |
| B03BB01 | folic acid | anti-anemic agent | 9541 | 9285 | 1.06 (0.99-1.14) |
| C09DA01 | losartan and diuretics | angiotension II receptor blockers | 1323 | 1270 | 1.06 (0.96-1.17) |
| C09AA02 | enalapril | ACE inhibitors | 14095 | 13426 | 1.07 (1.02-1.11) |
| C01AA05 | digoxin | cardiac glycosides | 6956 | 6647 | 1.07 (1.00-1.14) |
| N06AB06 | sertraline | anti-depressants | 2667 | 2521 | 1.07 (0.95-1.19) |
| S01BA01 | dexamethasone | anti-inflammatory agents | 1239 | 1186 | 1.07 (0.96-1.20) |
| C07AB02 | metoprolol | beta blocking agents | 24140 | 22771 | 1.07 (1.04-1.11) |
| A06AC03 | sterculia | anti-constipation drug | 680 | 625 | 1.08 (0.95-1.23) |
| A02BC01 | omeprazole | proton pump inhibitors | 17273 | 16203 | 1.08 (1.04-1.13) |
| C03CA01 | furosemide | diuretics | 29041 | 27956 | 1.08 (1.04-1.12) |
| N06AA09 | amitriptyline | antidepressants | 1401 | 1305 | 1.08 (0.94-1.25) |
| C01DA02 | glyceryl trinitrate | vasodilators | 3546 | 3102 | 1.09 (1.03-1.16) |
| H02AB06 | norethisterone and estrogen | sex hormones and modulators | 6122 | 5848 | 1.09 (1.02-1.17) |
| C03AA03 | hydrocholorthiazide | diuretics | 1219 | 1167 | 1.09 (0.97-1.23) |
| M01AB05 | diclofenac | anti-inflammatory agent | 2389 | 2214 | 1.10 (1.02-1.18) |
| J01CF05 | flucloxacillin | antibiotics | 1704 | 1499 | 1.12 (1.03-1.22) |
| J01CE02 | phenoxymethylpenicillin | antibiotics | 1445 | 1231 | 1.13 (1.04-1.23) |
| C07AA05 | propranolol | beta-blocking agents | 1060 | 1017 | 1.13 (0.98-1.31) |
| C03DA01 | spironolactone | diuretics | 5781 | 5407 | 1.13 (1.05-1.22) |
| A12BA01 | potassium chloride | mineral supplements | 4869 | 4590 | 1.14 (1.04-1.24) |
| N05BA01 | diazepam | anxiolytics | 2021 | 1931 | 1.14 (1.01-1.27) |
| N05BA04 | oxazepam | anxiolytics | 9180 | 8827 | 1.15 (1.08-1.23) |
| C02CA04 | doxazosin | antiadrenergic agents | 513 | 471 | 1.16 (0.97-1.39) |
| J01CA04 | amoxicillin | antibiotics | 848 | 682 | 1.17 (1.04-1.31) |
| N06AB04 | citalopram | antidepressants | 10597 | 10121 | 1.18 (1.10-1.27) |
| A06AD15 | macrogol | anti-constipation drug | 360 | 297 | 1.19 (1.00-1.42) |
| C07AB07 | bisoprolol | beta blocking agents | 8074 | 7193 | 1.20 (1.13-1.27) |
| N03AX16 | pregabalin | antiepileptics | 1440 | 1340 | 1.20 (0.99-1.44) |
| J01CA08 | pivmecillinam | antibiotics | 1380 | 1124 | 1.21 (1.11-1.32) |
| N06AB10 | escitalopram | antidepressants | 1179 | 1112 | 1.21 (1.00-1.47) |
| N02AE01 | buprenorphine | opioids | 949 | 843 | 1.22 (1.06-1.40) |
| B03AA07 | ferrous sulfate | antianemic drug | 4614 | 4235 | 1.23 (1.12-1.35) |
| R05FA02 | opium derivatives and expectorants | cough suppressants and expectorants | 1237 | 976 | 1.23 (1.12-1.35) |
| J01MA02 | ciprofloxacin | antibiotics | 1514 | 1153 | 1.23 (1.12-1.35) |
| J02AC01 | fluconazole | antimyotics | 337 | 262 | 1.24 (1.11-1.50) |
| A06AB08 | sodium picosulfate | anti-constipation drugs | 1111 | 783 | 1.24 (1.11-1.38) |
| D01AC03 | econazole | antifungals | 304 | 241 | 1.25 (1.03-1.51) |
| J01AA02 | doxycycline | antibiotics | 1224 | 951 | 1.26 (1.15-1-38) |
| B01AC04 | clopidogrel | antithrombotics | 2433 | 2067 | 1.27 (1.16-1.39) |
| B01AB05 | enoxaparin | antithrombotics | 251 | 196 | 1.27 (0.98-1.66) |
| J01XE01 | nitrofurantoin | antibiotics | 776 | 631 | 1.28 (1.13-1.45) |
| J01DB05 | cefadroxil | antibiotics | 303 | 229 | 1.28 (1.06-1.55) |
| A07AA02 | nystatin | antibiotics | 452 | 331 | 1.28 (1.08-1.51) |
| J01EA01 | trimethoprim | antibiotics | 814 | 658 | 1.28 (1.14-1.45) |
| N05AD01 | haloperidol | antipsychotics | 900 | 830 | 1.29 (0.99-1.66) |
| J05AB01 | aciclovir | antivirals | 155 | 113 | 1.29 (0.98-1.70) |
| R01AX03 | ipratropium bromide | decongestants and used in nasal preparations. For example, rhinitis treatment. | 195 | 163 | 1.31 (0.99-1.73) |
| B01AC07 | [dipyridamole](https://www.whocc.no/atc_ddd_index/?code=B01AC07) | antithrombotics | 731 | 574 | 1.33 (1.17-1.51) |
| N02AA05 | oxycodone | opioids | 4285 | 3571 | 1.34 (1.21-1.48) |
| N03AX09 | lamotrigine | antiepileptics | 643 | 566 | 1.34 (1.00-1.79) |
| R03AL02 | salbutamol and ipratropium bromide | adrenergics | 379 | 299 | 1.35 (1.06-1.72) |
| N06AX11 | mirtazapine | antidepressants | 6333 | 5897 | 1.36 (1.23-1.49) |
| A07EC02 | mesalazine | intestional anti-inflammatory agents | 221 | 195 | 1.36 (0.99-1.88) |
| H02AB01 | betamethasone | glucocorticoids | 1022 | 693 | 1.36 (1.21-1.54) |
| B03AA01 | ferrous glycine sulfate | antianemic drug | 345 | 286 | 1.37 (1.04-1.80) |
| N05AX08 | risperidone | antipsychotics | 2920 | 2806 | 1.43 (1.19-1.73) |
| N02CC01 | sumatriptan | anti-migraine drug | 199 | 151 | 1.45 (1.05-2.00) |
| B01AB04 | dalteparin | antithrombotics | 1155 | 807 | 1.45 (1.27-1.67) |
| N02AB03 | fentanyl | opioids | 809 | 696 | 1.45 (1.21-1.75) |
| A03FA01 | metoclopramide | anti-emetic and propulsives | 1009 | 669 | 1.46 (1.27-1.67) |
| A12BA02 | potassium citrate | mineral supplements | 134 | 95 | 1.46 (1.02-2.07) |
| S01BA02 | hydrocortisone | anti-inflammatory agents | 122 | 92 | 1.46 (1.06-2.01) |
| A04AA01 | ondansetron | antiemetics and antinauseants | 178 | 102 | 1.47 (1.06-2.04) |
| H03BB02 | thiamazole | antithyroid agent | 162 | 114 | 1.47 (0.95-2.27) |
| B01AB10 | tinzaparin | antithrombotics | 445 | 302 | 1.48 (1.19-1.84) |
| R06AE05 | meclozine | antihistamines | 154 | 98 | 1.51 (1.09-2.08) |
| M03BX01 | baclofen | muscle relaxants, centrally acting agents | 229 | 205 | 1.55 (0.96-2.51) |
| A06AG10 | docusate sodium, *incl.* combinations | anti-constipation drug | 68 | 39 | 1.57 (1.01-2.46) |
| N06DX01 | memantine | anti-dementia drug | 1212 | 1083 | 1.63 (1.28-2.07) |
| A02AD01 | ordinary salt combinations | antacids | 53 | 34 | 1.64 (1.03-2.59) |
| A02BD06 | esomeprazole, amoxicillin and clarithromycin | anti-ulcer drug | 71 | 37 | 1.64 (1.08-2.50) |
| N05CM02 | clomethiazole | hypnotics and sedatives | 1076 | 923 | 1.64 (1.33-2.03) |
| N02AA01 | morphine | opioids | 2533 | 1954 | 1.66 (1.46-1.88) |
| P01AB01 | metronidazole | antiprotozoals | 412 | 216 | 1.73 (1.43-2.10) |
| N02AA55 | oxycodone and naloxone | opioids | 208 | 62 | 2.05 (1.17-3.60) |
| A06AA01 | liquid paraffin | anti-constipation drug | 37 | 16 | 2.12 (0.95-4.74) |
| L02AA02 | polyestradiol phosphate | estrogens | 31 | 15 | 2.19 (0.91-5.27) |
| B01AF01 | rivaroxaban | antithrombotics | 83 | 35 | 2.39 (1.51-3.76) |
| A04AA03 | tropisetron | antiemetics and antinauseants | 40 | 17 | 2.39 (1.02-5.61) |
| B01AF02 | apixaban | antithrombotics | 51 | 25 | 2.41 (1.25-4.65) |
| B01AC24 | ticagrelor | antithrombotics | 122 | 50 | 2.96 (1.87-4.66) |
| N05CD08 | midazolam | hypnotics and sedatives | 18 | 4 | 3.19 (0.96-10.64) |
| A03AB02 | glycopyrronium bromide | muscarinic antcholinergic group | 31 | 4 | 3.38 (1.25-9.15) |
| N02AG01 | morphine and antispasmodics | opioids | 167 | 62 | - 1. 2.45-5.44) |

[All generic names listed according to the Anatomical Therapeutic Chemical (ATC), 5^th^ level

Case crossover analysis, case-period (1-30 days) and control period (61-90 days) before the index-date for the diagnosis of first ischemic stroke]
